# Molecularly Imprinted Polymers Combined with Electrochemical Sensors for Food Contaminants Analysis

**DOI:** 10.3390/molecules26154607

**Published:** 2021-07-29

**Authors:** Dounia Elfadil, Abderrahman Lamaoui, Flavio Della Pelle, Aziz Amine, Dario Compagnone

**Affiliations:** 1 Faculty of Bioscience and Technology for Food, Agriculture and Environment, University of Teramo, Via Renato Balzarini 1, 64100 Teramo, Italy; delfadil@unite.it (D.E.); fdellapelle@unite.it (F.D.P.); 2 Laboratory of Process Engineering and Environment, Faculty of Sciences and Techniques, Hassan II University of Casablanca, Mohammedia 28810, Morocco; lamaoui.abderrahman@gmail.com

**Keywords:** molecularly imprinted polymers, electrochemical sensors, pesticides, veterinary drugs, process and package contaminants, solid-phase extraction, food safety, electropolymerization

## Abstract

Detection of relevant contaminants using screening approaches is a key issue to ensure food safety and respect for the regulatory limits established. Electrochemical sensors present several advantages such as rapidity; ease of use; possibility of on-site analysis and low cost. The lack of selectivity for electrochemical sensors working in complex samples as food may be overcome by coupling them with molecularly imprinted polymers (MIPs). MIPs are synthetic materials that mimic biological receptors and are produced by the polymerization of functional monomers in presence of a target analyte. This paper critically reviews and discusses the recent progress in MIP-based electrochemical sensors for food safety. A brief introduction on MIPs and electrochemical sensors is given; followed by a discussion of the recent achievements for various MIPs-based electrochemical sensors for food contaminants analysis. Both electropolymerization and chemical synthesis of MIP-based electrochemical sensing are discussed as well as the relevant applications of MIPs used in sample preparation and then coupled to electrochemical analysis. Future perspectives and challenges have been eventually given.

## 1. Introduction

The increasingly high presence of contaminants in food is an alarming and critical issue that will put human health at risk. Indeed, in recent decades, population growth, globalization, and industrialization have led to the dissemination and increase in the variety and quantity of contaminants in the food chain. The common food safety problems can be divided into three categories: chemical contaminants (e.g., pesticides, veterinary drugs), microbial contaminants (e.g., bacterial, fungal, and viral contaminants), and physical contaminants (e.g., hair, plant stalks, or pieces of plastic metal or stone) [1,2].

The increase in the quantity and variety of food contaminants has led to more restrictive regulations and, therefore, to more demands on rapid food analysis. Determination of contaminants in food samples involves significant challenges, since the complexity of the matrix and the potential interferents. In this context, powerful analytical techniques (e.g., high-performance liquid chromatography and gas chromatography coupled to mass spectrometric detection) are used for the detection of contaminants in food. However, these techniques often are not fast and cost-effective as desired since they require complex and expensive instrumentation, laborious sample pre-treatment procedures, and long analysis times.

The growing demand for easy-to-use and inexpensive analytical devices [3,4,5,6] capable of rapidly providing valuable qualitative and quantitative information increases the interest in electrochemical sensors because of their reduced size, portability, low cost, and low reagent and sample consumption [7]. A robust electrochemical sensor may require the successful integration of a transducer (an electrochemical cell generally comprising a working electrode, a reference electrode, and a counter electrode) with a biological recognition element (e.g., antibody, protein, enzyme, etc.) or artificial one (e.g., MIPs). MIPs have gained attention as recognition elements for sensor development due to their high selectivity towards the target analyte and their advantages compared to biological receptors: (i) easy and low-cost preparation, (ii) physical and chemical robustness when unfavorable conditions are used, such as organic solvents, extreme pH values, high temperatures and/or high pressures, (iii) reusability, (iv) stability and (v) possibility of large scale production [8,9].

The basis of today’s molecular imprinting technology started with the studies presented by Wulff and Sarhan (1972) almost 50 years ago [10,11]. In the first decades, the development and application of MIPs were mainly focused on separation and extraction techniques. Nowadays, MIPs are used in a wide variety of applications, including sample preparation (e.g., solid phase extraction) [12,13] chromatographic separation [14], chemical sensing [15], and drug delivery [16]. 

Electropolymerisation has facilitated the application of MIPs in electrochemical sensing by employing electroactive monomers that can polymerize by applying an appropriate potential or performing potentials scans in a defined range [17,18]. Along with electropolymerization, the modification of electrodes with MIPs prepared by traditional methods such as bulk polymerization can be also explored. More recently, the MIP technology has been coupled to disposable and miniaturized screen-printed electrodes [19]. So, in the future, it is foreseen that some MIPs could be used in commercial sensing. Theoretically, MIPs can be prepared for any molecule of interest.

**Figure 1:**
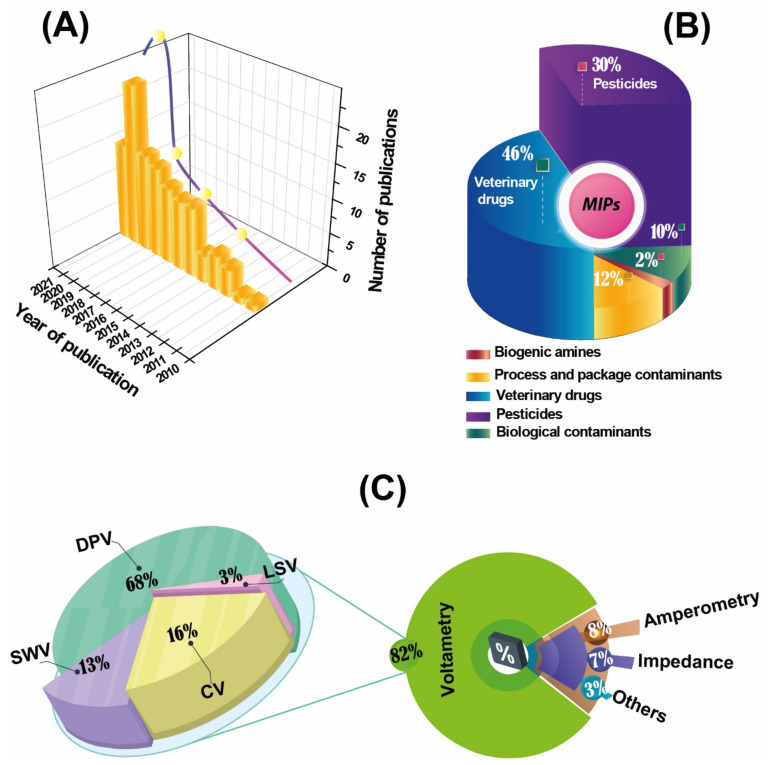
(**A**) Number of publications in the field of MIPs based electrochemical sensors for food safety monitoring between 2010 and 2021 (Consulted 15-04-2021) (www.mipdatabase.com accessed on 28 July 2021), (**B**) Application of MIPs based electrochemical sensors (MIPES) for food contaminants determination between 2010–2021. (**C**) Use of electrochemical techniques (amperometry, impedance, voltammetry, and others) as detection methods in combination with MIP sensor, and giving more focus on the use of voltammetry techniques (differential pulse voltammetry (DPV), cyclic voltammetry (CV), square wave voltammetry (SWV) and linear sweep voltammetry (LSV)) as detection methods in combination with the MIP sensor for food safety between 2010 and 2021 (Scopus database 14-06-2021).

To prepare this review, we have referred to the MIP database (http://mipdatabase.com accessed on 28 July 2021) that compiles MIPs reports for more than 10,000 target molecules as can be seen from Figure 1A; the development of MIP-based electrochemical sensors has exploded in the last decade while their applications for the analysis of food contaminants decreased.

Various review articles have been published on MIP [17,20,21]. However, to the best of our knowledge, there are very few review papers that cover the aspect of the use of MIP-based sensors for food safety [2,22]. Cao et al. [2] reported a review on MIPs based sensing strategies for the detection of food safety hazard factors covering the last five years (2014–2019). Interestingly, since 2012 there has been a steady increase in the number of articles published in the field of MIPs combined with electrochemical sensors for food analysis (Figure 1A), proving how this research field represents an evolving and expanding research hot topic.

This review aims to discuss the most exciting analytical features offered by coupling MIPs with electrochemical sensors in food analysis, covering the period 2010–2021 (Figure 1A), focusing particular attention on the sensors’ final applications and contaminants determination. In the first section, some generalities about MIPs and electrochemical sensors are reported. In the second section, the recent applications of MIPs-based electrochemical sensors and the combination of MIPs-based solid-phase extraction and electrochemical sensors for food analysis are presented and discussed. In the last section, general conclusions and future directions are highlighted.

## 2. Generalities about MIPs

### 2.1. General Concepts

**Figure 2:**
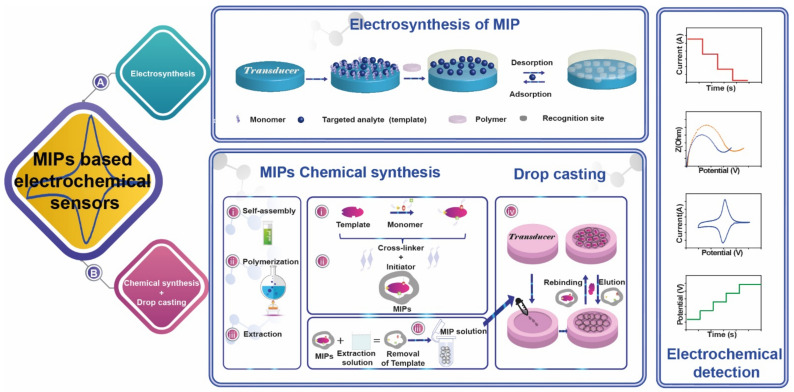
Scheme of the preparation of MIP-based electrochemical sensors, including the electrochemical and chemical synthesis.

MIPs, also known as “artificial antibodies“, have attracted the attention of researchers in the field of food safety due to their distinct advantages. The typical preparation of a MIP-based electrochemical sensor is displayed in Figure 2 for electrochemical (Figure 2A) and chemical synthesis (Figure 2B). The latter mainly includes four stages: (i) in a proper solvent, template molecule is preassembled with functional monomer to form a complex through covalent or non-covalent bond/interaction; (ii) after adding crosslinker, light or heat polymerization is carried out by an initiator, forming a crosslinked polymer; (iii) the imprinted molecules (template) in the polymer are eluted, leaving stable three-dimensional imprinted cavities that match the size and shape of the target molecule; (iv) drop-casting of the MIP solution onto the electrode surface. The “lock-key” process is the core of molecular imprinting, which significantly reduces the interference of non-template during recognizing process. For this reason, the technique has also been referred to as “host-guest” polymerization or “template” polymerization. Meanwhile, the types of monomer, crosslinker, solvent, and other polymerization elements are fundamental, since can affect the mass transfer rate, the number of effective imprinting cavities, and their matching degree with the targets [8,17].

### 2.2. MIPs Components Selection by Computational Approach

Recently, the computational approach has emerged as a potential route for the selection of the best configuration of chemicals for MIPs preparation. This strategy has also been applied to prepare MIPs for food analysis [23,24,25]. The principle of this approach is based on the theoretical calculation of the binding energy of the monomer-template (Δ*E*) according to the following equation:

Δ*E* = *E* _template-monomer_ − (*E* _monomer_ + *E* _template_) (1)

where Δ*E* is the binding energy (interactions energy). *E _template-monomer_*, *E _template_*, and *E _monomer_* are the energies of the template-monomer complex, molecule template, and functional monomer, respectively. These energies (*E _template-monomer_*, *E _template_*, and *E _monomer_)* can be obtained using a computational method such as Density Functional Theory (DFT) or Hartree–Fock. The DFT and Hartree–Fock are quantum computational methods for studying electronic structures [26]. Gaussian is among the most used software for this purpose. The selection of appropriate porogen solvent is also possible by varying the solvent during the calculations. The computational method is an excellent way to determine the most favorable combination for monomers and solvents and to understand the nature of the interaction between the functional monomer and template. This strategy allows reduction of the consumption of reagents, solvents, and times compared to a full “trial-and-error” approach [26].

## 3. Preparation of MIPs-Based Electrochemical Sensors

The first electrochemical sensor based on MIP was designed at the beginning of the 1990s by the Mosbach group [27]. Within three years, Hedborg and his co-authors reported the first thin molecular imprint based on a polymer membrane. In this case, the membrane was composed of aniline with the imprinted sites for l-phenylalanine. The MIP was used as a film onto a field-effect capacitor [28].

Conventionally, two different strategies are pursued to combine MIPs, and electrochemical sensors: prepare MIP directly on the electrode surface through electropolymerization (Figure 2A) and prepare MIP and deposit it on the electrode surface (Figure 2B). The latter can be achieved via drop-casting/coating or by magnetic capture onto magneto-actuated electrodes, taking advantage of MIPs synthesized onto magnetic particle.

### 3.1. Preparation of MIP onto Transducers Surface by Electropolymerization

MIPs formation assisted by electropolymerization, shown in Figure 2A, is typically conducted by applying a suitable potential or performing potentials scans in a solution containing the template, a proper functional monomer, and a porogen solvent in contact with the transducer surface. This approach has been claimed to be very efficient since it allows the control of the thickness of the polymer layer onto the transducer surface and does not require an external initiator to start the reaction (i.e., polymerization). Besides, using this strategy it is possible to prepare MIPs for templates that are thermo-sensitive and soluble in an aqueous medium such as proteins, bacteria, and viruses. Electropolymerization allows production in short times of MIPs with high retention capacity and with fast uptake kinetics, allowing to reach easily nanomolar limits of detection with high selectivity.

### 3.2. Modification of Transducers Surface by Chemically Synthetized MIPs

**Figure 3:**
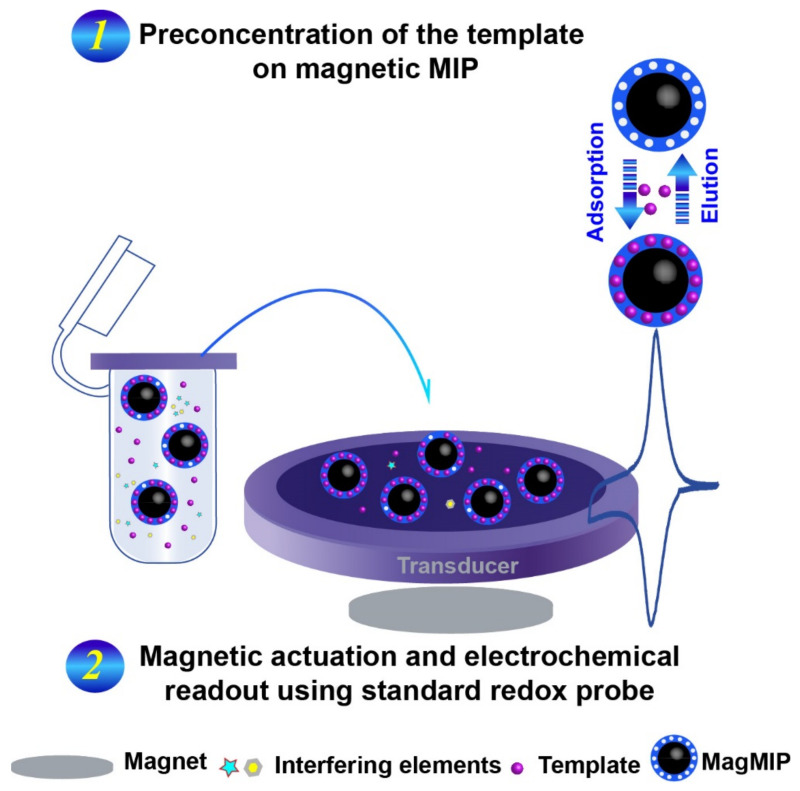
Schematic procedure for the electrochemical sensing of a target analyte preconcentrated on MagMIPs.

Two main mechanisms of polymerization may be distinguished, such as condensation and radical polymerization [2,29]. The latter is the most used method to prepare MIPs because it can be carried out in a wide range of temperatures. Using radical polymerization, it is possible to prepare MIPs in different formats, such as bulk form or nano/micro dispersions; there are many functional monomers with different functional groups that can be employed with this strategy. According to the initiation step, different approaches have been described, as thermal heating [30,31], microwave [32,33], ultrasound [34], photosynthesis [35], etc. After the MIPs synthesis, the removal of the template involves the use of an appropriate solution that has the potential to elute the template without affecting the holding polymer. Finally, the MIP can be deposited onto the surface of the transducer using drop-casting/coating strategies as reported in Figure 2B. Recently, the use of magnetic recognition elements for the electrochemical detection of various types of compounds has been considerably improved as a new detection strategy. This detection strategy is mainly based on the use of an external magnet to attract magnetic nano/microparticles covered with (bio)recognition elements onto an electrode/transducer surface. This strategy has also been used for magnetic molecularly imprinted polymers (MagMIPs), mainly chemically prepared. MagMIPs attraction from the ‘sampling solution’ (containing the analyte) to the transducer surface allows selective extraction from complex matrices and concentration of the analyte (Figure 3) [29,36].

Figure 3 shows a graphical sketch of MagMIPs in combination with an electrochemical transducer.

## 4. Electrochemical Techniques Combined with MIP-Sensors

In electrochemical sensors, the detection of the analyte is based on the variations at the electrode surface in terms of alteration in current and or voltage. According to the analytical signal output, the measurement is the potential (V) in potentiometry, the current (A) in voltammetry and amperometry, the resistance (Ω) in impedance, and Siemens (S) in the conductance method [37].

The use of these electrochemical techniques as detection methods combined with the MIP sensor is in the order of 82%, 8%, and 7% for voltammetry, amperometry, and impedance spectroscopy, respectively (Figure 1C). Voltammetry is the most used because of its high sensitivity, simplicity, and fast response.

The choice of measurement technique is generally guided by the electrochemical properties of the target molecule. Electroactive targets can be quantified by the current generated in amperometry or voltammetry. Non-electroactive targets may cause a change in the MIP film conductivity and/or porosity. This change could be controlled and monitored indirectly using CV, conductimetry, or electrochemical impedance spectroscopy (EIS) using an external redox probe [17]. Redox probes such as Ferri/ferrocyanide, hexaammine ruthenium chloride, or ferrocene are the most commonly used in combination with MIPs for indirect detection because of their fast charge transfer for several modified and/or unmodified transducers. The rebinding, at different concentrations, of a non-electroactive target analyte to an MIP, reduces proportionally the porosity of MIP film and the permeability of the redox probes to the electroactive sites, which subsequently results in a decrease of the current intensity/increase of the sensor impedance. This approach can therefore be used to quantify the analyte indirectly. Besides, morphological changes in the polymer itself may occur due to a specific interaction, resulting in changes in the diffusion rate of the redox probe, which can be recorded as a change in the faradic current [38].

**Figure 4:**
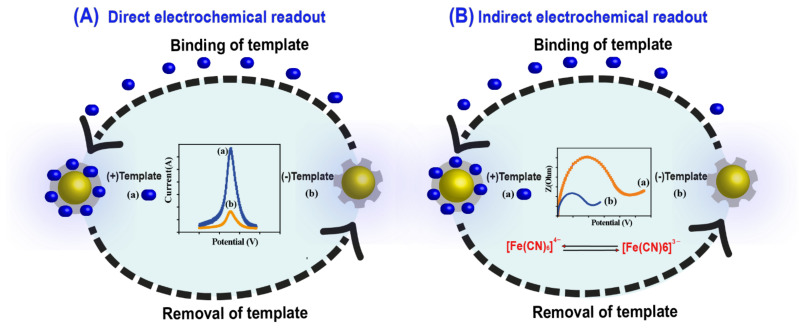
Graphical sketch of generic (**A**) direct and (**B**) indirect electrochemical measurements combined with MIPs.

Figure 4 illustrates the general concept of direct and indirect transduction systems combined with MIP. In general, the measurements with MIP-based electrochemical sensors take place with a three-step procedure, including the rebinding of the target analyte, the electrode washing to remove the non-specific binding, and finally, the electrochemical measurement.

### 4.1. Detection by Voltammetry

Voltammetry is a group of electroanalytical techniques working at a controlled variable potential. The current is measured as a function of applied potential. Indeed, for any analyte, as long as it can be reduced or oxidized electrochemically, the resulting faradic current is proportional to its concentration. The advantages of voltammetry are selectivity and sensitivity with a broad concentration range towards several analytes, fast analysis times (few seconds), and the ability to determine kinetic parameters and estimate the mechanisms of chemical and/or electrochemical reactions [39].

The most applied voltammetric techniques are differential pulse voltammetry (DPV), cyclic voltammetry (CV), square wave voltammetry (SWV), and linear sweep voltammetry (LSV).

The DPV technique is one of the most used voltammetric techniques in the field of MIP-based sensors due to its simplicity and high sensitivity, and the ability to reduce the noise generated by capacitive currents.

### 4.2. Detection by Amperometry

Amperometric techniques are generally run under a stirred solution or in a flow system since the mass transport of electroactive species should be governed only by convection. Amperometry allows the detection of an analyte by measuring the current at a constant applied potential, relating the measured current to the analyte concentration [40]. In MIP-coated electrodes, the signal in amperometric devices depends on the mass transfer rate of electrochemically active analytes into MIP film [3]. MIP-based amperometric sensors are simpler to use and may be easily integrated into microfluidic systems for continuous and real-time analysis.

### 4.3. Detection by Electrochemical Impedance Spectroscopy

The EIS technique is simple, inexpensive, and fast. “Impedance” refers to a physical variable that examines the characteristics of the resistance of an electrical circuit in the presence of an alternating current applied between the electrodes. In this system, the current flow response is measured by applying a small sinusoidal potential and detecting changes in frequency (f) from the applied potential over a wide frequency range. EIS can assess the intrinsic material properties and investigate the particular processes involved in the conductivity/resistivity or the capacitivity of the electrochemical system. These systems serve as very useful tools for the characterization and analysis of materials and biosensor transductions [41].

The charge transfer resistance (Rct) values change upon the stepwise modification/passivation of the electrode surface. Rct increasing indicate that the electron transfer became more complex at the sensor surface [42]. Besides impedance, direct measurement of conductivity has been also reported in the realization of a MIP-based atrazine sensor [43].

### 4.4. Detection by Potentiometry

In potentiometry, change in potential, evaluated under flow or batch conditions is used to evaluate or quantify the amount of an ion in solution. A potentiometric sensor operates under conditions of near-zero current flow. It measures the difference in potential between the working electrode and a reference electrode. Selectivity is introduced through membranes formed from MIPs containing specific ion exchangers or neutral carriers. Devices can also include chemically sensitive field-effect transistors. These semiconductor devices respond to the surface electric gradient or charge at the gate electrode [44]. The binding of the analyte by the MIP at the surface of the silicon chip shifts the surface potential., affecting the current, which allows the rate of the reaction to be monitored [45].

## 5. MIP-Based Electrochemical Sensors

### 5.1. Chemically Synthesized MIPs

In this section, we report the use of MIP chemically synthesized and later assembled onto electrochemical sensors for the detection of various chemical and biological hazards, sub-sections are divided according to the target analyte class.

#### 5.1.1. Pesticides

Different methods have been employed to quantify pesticides. Although HPLC coupled to mass spectroscopy represent the reference method [46] different sensoristic approaches have been attempted taking advantage of biosensors [47], nanomaterials-based sensors [48], and electrophoretic chips [49,50]. Pesticide sensing with a high level of selectivity and sensitivity plays an important role in food safety assessment [51,52].

MIP coupled to sensors for pesticides detection can help to overcome the limitations of traditional analytical approaches, particularly for the high selectivity, offering an excellent opportunity to develop practical, low-cost, and rapid detection methods [51,53,54,55,56,57].

Table 1 summarizes the research papers so far published on chemically synthesized MIPs-based electrochemical sensors for the analysis of contaminants. The most important parameters affecting the polymerization of MIPs and the analytical performance, such as the target analyte, the monomer, the extraction solution, the electrochemical technique, the linear range, the limit of detection (LOD), and the application in the real sample are reported.

A sensitive and selective MIP-modified screen-printed gold electrode (Au-SPE) was developed to monitor Malathion (MAL), in olive fruits and oils. The MIP sensor was prepared using acrylamide as the functional monomer, bisacrylamide as a cross-linking monomer, and MAL as the template molecule. Good linearity was obtained to bind MAL in the concentration range of 3 × 10^−16^–3 × 10^−12^ mol L^−1^. LOD of 2 × 10^−16^ mol L^−1^ was obtained using DPV [58].

An ionic liquid–graphene composite film coated glassy carbon electrode (MIP-IL-Gr/GCE) was used for the quantification of methyl parathion (MP). Initially, the MIP was prepared by free radical polymerization using methacrylic acid (MAA) as a functional monomer, ethylene glycol dimethacrylate (EGDMA) as a cross-linking agent, and 2,2′-azobis(isobutyronitrile) (AIBN) as initiator. Selective and sensitive detection of MP with a LOD of 6 × 10^−9^ mol L^−1^ was achieved using CV. The MIP developed was successfully applied for monitoring the quality of cabbage and apple peel samples [53].

Magnetic NPs (MNPs) have also been widely used to synthesize MIPs based on electrochemical sensors. This material can be manipulated by magnetic fields that can be applied in the preconcentration for the detection of targets in complex samples. Moreover, MNPs can be integrated on the surface of magneto-electrodes for electrochemical reaction readout using permanent magnets. Li et al. [59] proposed a MIP sensor for the detection of the herbicide chlortoluron based on the field-effect transistor. The MIP was synthesized on the surface of magnetic nickel (II) oxide (NiO) nanoparticles. Chlortoluron can be detected indirectly by the decrease in H_2_O_2_ oxidation current on the NiO nanoparticles modified GCE induced by the access blockage after re-binding. Good sensitivity was achieved due to the high catalytic effect of NiO nanoparticles on H_2_O_2_ oxidation, with a LOD of 2 × 10^−9^ mol L^−1^. Water samples were analyzed using the MIP sensor, and recoveries from 97% to 105% were obtained. Another research group also proposed a magnetic nickel hexacyanoferrate (NiHCF) nanoparticle-coated MIP sensor for the determination of chlorotoluron [60]. Here, the indirect determination of chlorotoluron was based on the change of current resulting from the oxidation of hydrazine hydrate on the modified electrode. This method is suitable for trace analysis of chlorotoluron because of (a) the advantage of the gate-controlled electro-catalytic amplification effect and (b) the enrichment of the analyte on the magnetic particles. The developed sensor showed that it could be reused several times. Water samples were spiked with chlorotoluron and recoveries obtained were ranging from 97% to 105% with a very low LOD of 9 × 10^−10^ mol L^−1^.

The exploration of new nanocomposites containing AuNPs and graphene with imprinted polymers offers an enormous number of imprinted cavities, increased surface area, admirable catalytic oxidation for the target analytes, and enhancement of physical properties. Recently, a MIP composite for carbamate pesticides combining reduced graphene oxide and gold nanoparticles (rGO-AuNPs) was developed. MIP was synthesized chemically using carbofuran as a template, methyl acrylic acid as a monomer, ethylene glycol maleic resinate acrylate as cross-linker, and toluene as porogen solvent. The obtained MIP suspension was deposited on the surface of an rGO-AuNPS/GCE sensor by drop-casting. The sensor had a LOD of 2 × 10^−8^ mol L^−1^ and good selectivity for the determination of carbofuran [61].

Graphene and a new cross-linker (ethylene glycol maleic resinate acrylate) for preparing sensitive molecularly imprinted sensors were proposed for phoxim determination by Tan et al. [62]. A MIP film was created on a graphene-modified GCE for the determination of phoxim using a free radical polymerization method. DPV was used as an electrochemical technique, and the obtained LOD was 2 × 10^−8^ mol L^−1^. The imprinted electrochemical sensor was employed to determine phoxim in cucumber samples with recovery ranging from 98% to 101%.

Amatatongchai et al. [63] proposed a facile method for the selective measurement of profenofos (PFF) using a simple flow injection system with a MIP-coated carbon nanotube amperometric sensor. The MIP-CNTs material was synthesized by coating the surface of the carboxylated CNTs with SiO_2_ and vinyl end groups and then terminating with MIP shells. The MIP was grafted onto the cores of the CNTs using MAA as a monomer, (EGDMA) as a crosslinking agent, and AIBN as an initiator. The PFF sensor was constructed by coating the surface of a GCE with 3D-CNTs@MIP followed by the extraction of the template. The imprinted sensor was applied to the detection of PFF in vegetable samples with a LOD of 2 × 10^−9^ mol L^−1^.

A simple imprinting route synthesis based on graphene (GN) was proposed by Zhang et al. [64] to fabricate an electrochemical sensor for sensitive and selective determination of imidacloprid (IDP) residue. The vinyl-functionalized graphene-decorated MIP was first synthesized by converting graphite to graphene (GN), then the GN was modified by *p*-vinyl benzoic acid (VBA) for two hours at room temperature, the template (IDP) was added to the GN-VBA mixture under stirring to form a complex of template molecule and functional monomer via interfacial hydrogen bonding. Later the crosslinker (EGDMA) and the initiator (AIBN) were added. The polymerization reaction was carried out at 60 °C for 24 h. The resulting GN-VBA-MIP was drop-cast onto the GCE surface with chitosan (CS). Removal of the molecules from the matrix was performed electrochemically by CV until no imidacloprid signal was present. Brown rice was used as a real sample, and a LOD of 1 × 10^−7^ mol L^−1^ was achieved.

Abdel-Ghany et al. [65] described a potentiometric sensor based on MIPs for the determination of the neonicotinoid insecticide dinotefuran in cucumber and soil samples. The MIP was synthesized by bulk polymerization using Acrylamide, EGDMA, AIBN, and dinotefuran as a monomer, crosslinker, initiator, and template molecule, respectively. A detection limit of 1.7 × 10^−6^ mol L^−1^was achieved.

A DFT-based computational method for the rational design of MIPs for the herbicide cyanazine (CZ) was introduced by Gholivand et al. [66]. The DFT revealed that acrylamide/toluene was the best combination of functional monomer/porogen solvent, leading to stable CZ-acrylamide complexes. The MIP worked as both a pre-concentrator and a highly selective recognition element in the carbon paste electrode. The authors used the stripping voltammetric (SV) technique, and achieved a LOD of 3 × 10^−9^ mol L^−1^ Another group also proposed a DFT calculation for MIP-modified CPE for the determination of hexazinone herbicide in river water samples; from this study, it is clear that the appropriate monomer depends mainly on the template nature. The simulations were performed to evaluate the interaction of hexazinone with twenty monomers. Lower calculated energy is associated with higher affinity and the resulting polymer should be more selective. Three functional monomers based on the theoretical data were used in MIP preparation, 2-vinyl pyridine, MAA, and acrylamide. The 2-vinyl pyridine-based MIP demonstrates high selective binding property toward the template hexazinone herbicide [67]. In this sense, DFT makes MIP synthesis more economical by reducing the number of trials and saving on reagent consumption.

#### 5.1.2. Veterinary Drugs

The amount of applications of MIPs for the determination of veterinary drugs in the period 2010–2021, is higher than for the other contaminants in as reported in Figure 1B.

Veterinary drugs are a potential group of chemical contaminants because they are designed, like other drugs, to have biological effects at low concentrations. They include not just the parent form of the chemical, i.e., active compound or prodrug (inactive precursor that is converted into an active form by normal metabolic processes), but also their bioactive metabolites and transformation products. Veterinary pharmaceuticals belong to several pharmacological categories: antimicrobials (antibiotics including growth promoters and antiseptics), antiparasitics (ectoparasiticides, endectocides, endoparasiticides including antiprotozoals, and anthelmintics), hormones, antifungals, anti-inflammatory drugs (steroidal and non-steroidal), anesthetics, euthanasia products, tranquilizers, sedatives, bronchodilators, antacids, diuretics, emetics, and emulsifiers [86,87].

In brief, antibiotics can be classified into seven main groups: tetracyclines, macrolide antibiotics, aminoglycosides, peptide antibiotics, lincosamides, streptograminsβ-lactam antibiotics, etc. Antibiotics can accumulate in the human body via the food chain, which can negatively influence human health, even at low concentrations. Besides, the overuse of antibiotics, related to both intensive farming and auxinic uses, has increased the frequency of resistance genes, leading to a decrease in the effectiveness of disease treatment. Moreover, even non-standardized legislation among Europe and other countries regarding their use represents a critical issue. Therefore, it is highly desirable to develop sensitive, selective, and straightforward methods for the rapid assessment of antibiotic levels in food samples [86].

Numerous studies have been reported about MIP and nanoparticles to achieve high sensitivity and rapid response to antibiotics. Recently, Surya et al. [68] proposed an electrochemical biomimetic sensor to detect ciprofloxacin (CIP). An AuNPs-chitosan decorated MIP (Ch-AuMIP) was used to modify GCE for the preparation of the sensor. The synergistic combination of AuNPs and Ch- MIP helps to determine CIP (LOD = 2 × 10^−7^ mol L^−1^) in different samples (water, mineral water, milk, and pharmaceutical formulation) with a satisfactory recovery in the range of 94 to 106% even in the presence of other analog molecules as Norfloxacin and Ofloxacin.

Liu et al. [69] proposed a self-supported Fe_3_N-Co_2_N (iron nitride- cobalt nitrides) nanoarray with high electrical conductivity and a large surface area growth on MIPs, further realizing a sensitive and stable electrochemical sensor for ampicillin detection. Firstly, Fe_3_N-Co_2_N/carbon cloth was prepared by the hydrothermal method. Secondly, MIPs was obtained by in situ polymerizations on the surface of the Fe_3_N-Co_2_N/carbon cloth electrode. The as-prepared MIPs electrochemical sensor allowed the detection of ampicillin with a low LOD (4 × 10^−10^ mol L^−1^) and exhibited good reproducibility and stability.

Long et al. [70] developed a magnetic imprinted electrochemical sensor based on magnetic multi-walled carbon nanotubes (MWCNTs) for the sensitive determination of kanamycin in complex matrixes as liver and milk. The MIP was prepared on the surface MWCNT-Fe_3_O_4_ nanoparticles using kanamycin as the template molecule and MAA as the functional monomer. A LOD of 2 × 10^−11^ mol L^−1^ was obtained using DPV.

Jafari et al. [71] developed an electrochemical MIP-nanosensor for Cloxacillin (CLO). Indeed, the electrochemical nanosensor was developed using a screen-printed carbon electrode (SPCE) modified with the MIP/graphene oxide (GO)/AuNPs nanocomposite direct detection of CLO from aqueous and milk samples. The pre-concentration technique leads to a LOD of 4 × 10^−10^ mol L^−1^.

Huang et al. [72] developed, through a sol-gel method, a molecularly imprinted electrochemical sensor with reduced graphene oxide and titanium dioxide modified platinum (Pt) electrode surface for the detection of toltrazuril (anticoccidial veterinary drug) in chicken muscle and egg. The synergistic fast electron transfer ability, large electroactive surface area, and high catalytic activity of rGO and TiO_2_ contribute to amplifying the electrochemical signal and, consequently, improving the sensor’s sensitivity. Using DPV, the electrochemical sensor displayed a wide linear concentration range from 1 × 10^−3^ to 1 × 10^−1^ mol L^−1^, with a LOD of 5 × 10^−4^ mol L^−1^.

Sulfonamides are a family of antibiotics used in human and veterinary medicine. Wie et al. [73] proposed an electrochemical sensor based on a GCE modified with MIP and graphene oxide (GO) for sulfanilamide. The MIP/GO material was prepared by precipitation polymerization at 60 °C for 24 h in the presence of sulfanilamide as a template molecule and MAA as a monomer. SWV was used as an electrochemical technique and applied to quantify sulfanilamide in milk with a LOD of 6 × 10^−10^ mol L^−11^.

Guo et al. [74] used hemin/graphene hybrid nanosheets (H-GNs) to initiate the imprinted polymerization by catalyzing the generation of free radicals using sulfamethoxazole (SMX) as the template and MAA as the monomer. Thus, MIPs using sulfamethoxazole as the template was directly prepared on the surface of H-GNs without any film modification. Most importantly, the template could be adsorbed on the H-GNs to enhance the number of imprinted sites and thus improve the selectivity of MIP films. The LOD was 5 × 10^−12^ mol L^−1^. This sensor was free from interference caused by analogs of sulfamethoxazole, which represent a novel insight for the preparation of the MIPs-based sensor for food safety monitoring.

Hormone agents such as estradiol are widely used in veterinary medicine [86]. Han et al. [75] fabricated a magnetic molecularly imprinted sensing film (MMISF) for the determination of estradiol (E2). The MagMIPs were synthesized by in situ polymerizations of glutathione (GSH)-functionalized gold (Au)-coated Fe_3_O_4_ (Fe_3_O_4_@Au-GSH) nanocomposites and aniline. The MMISF was constructed with MagMIPs via a soft modification on the surface of a magnetic GCE(MGCE) or removed from it by freely installing a magnet into MGCE. The MMISF was used for detecting E2 in milk powder with good sensitivity, selectivity, and reproducibility, and the LOD achieved was 3 × 10^−9^ mol L^−1^.

Bai et al. [76] developed a MIP based on AuNPs/MWCNTs-Chitosan composites electrochemical sensor for diethylstilbestrol (synthetic estrogen and as a growth promoter). The MIP was prepared through the sol-gel method. AuNPs and an MWCNTs chitosan composite (MWCNTs-Chitosan) were stepwise used to modify a GCE. These two nanomaterials were used to increase the electrode surface area to enhance the electron transfer rate and amplify the sensor signal. The MIP was electrodeposited on the electrode surface. The obtained results demonstrated an extremely low LOD 9 × 10^−17^ mol L^−1^ using DPV.

#### 5.1.3. Process and Package Food Contaminants

Undesirable chemical compounds in food can be generated during processing by chemical reactions between food constituents and/or ingredients. They can also be released from materials, machinery, containers, and even packaging. For example, when a food is processed by heat (baking, frying, etc.) reactions occur between the components of the food; some of these interactions can lead to the formation of undesirable compounds (e.g., acrylamide). Similarly, for instance, food storage in plastic can cause the release of undesirable chemical compounds. Phthalates and bisphenol A are the most ubiquitous chemicals that are used as a plasticizer in almost every plastic product manufacturing, including toys, food packaging, and pharmaceutical consumables. The teratogenic and carcinogenic effects of these synthetic chemicals have been well-cited in published research as a possible risk to the health of all living organisms on earth [88,89]. Unfortunately, phthalates detection techniques require expensive equipment, extended analysis time, and result in very complex procedures.

Zhao et al. [82] proposed a GCE MIP-based sensor for Diisononyl phthalate (DINP). The electrode was assembled by mixing MIPs and agarose. The MIPs-modified electrode can detect the analyte in the sample without sample pretreatment. A LOD of 3 × 10^−10^ mol L^−1^ in a white liquor sample was obtained using direct detection by CV.

Li et al. [89] proposed a composite of Magnetic Graphene Oxide Gold Nanoparticles-(MGO-AuNPs) and MIPs for the determination of dibutyl phthalate. The composite of MGO@AuNPs was first synthesized using coprecipitation and self-assembly techniques. Subsequently, the template molecules (DBP) were absorbed onto the surface of MGO-AuNPs due to their excellent affinity. Then the selective copolymerization of MAA and ethylene glycol dimethacrylate was performed on the surface of MGO@AuNPs. The electrochemical sensor of DBP showed good repeatability for 30 repeated analyses and low LOD (8 × 10^−10^ mol L^−1^) using DPV as the electrochemical technique.

Deng et al. [90] proposed graphene flakes doped chitosan film as imprinted sensing nanocomposite on an acetylene black paste electrode (ABPE) for bisphenol A (BPA) detection in plastic bottled drinking water and canned beverages. The achieved LOD was 6 × 10^−9^ mol L^−1^ using derivative voltammetry.

Huang et al. [78] proposed MWCNTs and AuNPs for the enhancement of electrons exchange and sensitivity. Thin-film of MIP prepared by sol-gel method was cast on a gold electrode by electrochemical deposition. They demonstrated that the developed sensor exhibited specific binding sites for BPA. with a LOD (LOD) of 4 × 10^−9^ mol L^−1^. The sol-gel technique offered an attractive route to enhance the stability of the imprinted sensor.

Dadkhah et al. [79] developed a GCE modified with amino-functionalized graphene oxide and MIP for electrochemical sensing of BPA. A facile procedure accomplished the functionalization of GO with amino groups with 3-aminopropyltriethoxysilane (APTES). Then, the template was immobilized onto amino-functionalized GO to improve the recognition ability of MIP-based sensors. Prior to polymerization, ethylene glycol dimethacrylate was also grafted onto the APTES coated graphene oxide sheets by the Michael addition reaction. In this way, many homogeneous imprinting sites were formed on the GO sheets. The resulting composite was placed on a GCE used to determine BPA directly by DPV. A LOD of 3 × 10^−9^ mol L^−1^ in milk and mineralized water without any pre-treatment and matrix interfering effects were detected.

Xu et al. [80] proposed the applications of graphene (Gr) modified acetylene black paste (ABPE) for BPA detection in a plastic pacifier. (4-Vinylpyridine/EGDMA/AIBN was used as MIP synthesis components. The MIPs were drop cast onto Gr/ABPE followed by IR lamp heating to remove the solvent. Fe(CN)6]^3–/4–^ was used as an electrochemical probe to study the response of BPA on the prepared electrode using DPV the LOD was 2 × 10^−13^ mol L^−1^.

Zhang et al. [81] developed a polymeric membrane potentiometric sensor using a soluble MIP (s-MIP) as a receptor. The s-MIP is synthesized by the swelling of the traditional MIP at a high temperature using methacrylic acid, divinylbenzene, and free-radical initiator AIBN as a monomer, cross-linker, and initiator, respectively. The obtained MIP can be dissolved in the plasticized polymeric membrane for homogeneous binding of the imprinted polymer to the target molecules. By using bisphenol, A as a model, the method exhibited an improved sensitivity compared to the conventional MIP-based sensors with a detection limit of 6 × 10^−8^ mol L^−1^. Moreover, the present sensor exhibits a good selectivity over other phenols. The proposed s-MIPs can provide an appealing substitute for the traditional insoluble MIP receptors in the development of polymeric membrane-based electrochemical sensors.

#### 5.1.4. Biogenic Amines

Consumption of food containing high levels of biogenic amines (BAs) has been associated with health hazards. Histamine, putrescine, tryptamine, cadaverine, tyramine, b-phenylethylamine, spermidine, and spermine are considered the most important biogenic amines in foods. Analysis of BAs with high specificity and selectivity is essential for their toxicity and usage as indicators of the degree of freshness or spoilage of food [21,22]. Recently, MIP coupled electrochemical sensors for BAs determination in food were investigated and applied successfully to real food matrices.

Tyramine is a BA produced by the decarboxylation of tyrosine. It is found widely in fermented foods and beverages, meat, fish, and other dairy products. Huang et al. [83] used the combination of MWCNT-gold nanoparticle (MWCNT-AuNP) composites and chitosan polymer to design MIP on a sol-gel matrix for the recognition of tyramine in yogurt samples. The MIP was synthesized using tyramine as the template molecule, silicic acid tetracthyl ester and triethoxyphenylsilane as the functional monomers and EGDMA as a cross-linking agent. The tyramine-MIP solution was deposited onto the MWCNT-AuNP/chitosan/GCE surface by drop-casting. Chitosan is acting as a bridge for the imprinted layer, while MWCNT-AuNP composites were introduced for the enhancement of conductivity and, then, the sensitivity of the electrode. A LOD of 6 × 10^−8^ mol L^−1^ was obtained using direct amperometric measurement.

Histamine is another biogenic amine from the decarboxylation of the amino acid histidine, catalyzed by the enzyme *L*-histidine decarboxylase [21]. Histamine is occurring in food and body fluids in harsh environments (acidic/basic environments, organic solvents, etc.). Therefore, the development of a robust analytical strategy that can support extreme pH conditions is necessary. Bongaers et al. [84] designed an impedimetric MIP-based sensor for detecting low amounts of histamine. Bulk polymerization was used for the synthesis of MIP. The developed MIP-sensor for histamine demonstrated its stability in a wide range of pH 5–12, showing good sensitivity and LOD of 2 × 10^−9^ mol L^−1^ and no interference response toward histidine.

#### 5.1.5. Bacterial Contaminants

Recently, Jiang et al. [85] designed a Fe_3_O_4_@SiO_2_@MIP-based voltammetric sensor for N-acyl-homoserine-lactones (AHLs) monitoring in clinical and food analysis. AHLs are markers of gram-negative bacteria present in food. For the synthesis of MagMIPs, MNPs were prepared using a solvothermal method, then modified sequentially with TEOS and APTES to introduce amine groups followed by surface polymerization using MAA, EGDMA, and AIBN as a monomer, crosslinker, and initiator, respectively. The MagMIPs were deposited onto a magnetic carbon paste electrode (MGCE) surface and characterized by direct electrochemical measurements. A LOD of 8 × 10^−10^ mol L^−1^ was achieved by DPV. The combination of the MIP with MNPs allows for a controllable re-binding process and enables easy, rapid magnetic separation and simple electrode regeneration. The quantitative detection of AHLs can be performed on bacterial supernatants in a more convenient, easier, and less expensive way than most available AHL detection methods.

Chemically synthesized MIPs for biological hazards, including bacteria, viruses, and parasites, are still in their preliminary stage and present a significant challenge due to the high sensitivity/biodegradability of biological targets to the demanding conditions of chemical synthesis. Electrochemically synthesized MIPs remains the preferred strategy for the development of whole-cell MIP electrochemical sensors.

### 5.2. Electropolymerised MIPs-Based Sensors

MIPs can be synthesized via electropolymerization, a reaction in which a monomer is generally oxidized onto the working electrode surface, giving rise to a polymeric film [91]. The presence of the target template in the electropolymerization medium usually results in the production of MIPs cavities after removal of the template. The electrosynthesized polymer can be easily controlled, particularly in thickness, by controlling the experimental conditions such as the number of voltammetric cycles or time of polymerization, applied voltage, and concentration of the monomer. In this way, a uniform coating, in a short time, is generally achieved with significant advantages over classical synthetic routes for MIPs [29]. Electrosynthesized MIP-based sensors for monitoring food contaminants received significant attention during the last years to detect chemical and biological hazards in food.

For a satisfactory application of MIPs in sensors, it is essential to reduce analysis time, improve binding kinetics and achieve complete template removal. As previously reported, the integration of nanomaterials such as AuNPs, nanocomposites such as chitosan-AuNPs into sensing layers can solve these issues by enhancing the sensor surface area, which in turn should increase the sensitivity of imprinted polymers.

Table 2 summarizes the research papers so far published on electrosynthesized MIPs-based electrochemical sensors for the analysis of contaminants. The most important parameters affecting the electropolymerization of MIPs and the analytical performance, such as the target analyte, the monomer, the extraction solution, the electrochemical technique, the linear range, the LOD, and the application in the real sample are reported. Veterinary drugs, pesticides, and mycotoxins are the most relevant targets that have been studied.

#### 5.2.1. Pesticides

Organophosphorus compounds constitute an important class of pesticides whose toxicity arises from inhibiting the acetylcholinesterase enzyme. Chlorpyrifos (CPF) is an organophosphate pesticide and is one of the most extensively used in agriculture, household, and urban insecticide applications [92]. Roushani et al. [93] proposed a highly sensitive electrochemical aptasensor based on the use of a GCE modified with an electropolymerized DNA aptamer of 91 bases-imprinted polymer and gold nanorods (AuNRs) for the quantification of CPF. The sensor was constructed by modifying a GCE with AuNRs, then with MIP using Ortho-phenylenediamine and O-dihydroxybenzene as functional monomers and CPF as template molecule and aptamer. The exploitation of AuNRs improved the electron transfer rate and provided a high surface area for immobilization of the MIP-aptamer. The detection limit was 3 × 10^−16^ mol L^−1^ using DPV. This is lower than any of the previously reported methods. This MIP-aptasensor is selective over structural analogs, stable, and adequately reproducible. It was successfully applied to the determination of CPF in spiked lettuce and apple food samples. Uygun et al. [92] proposed an impedimetric sensor based on a molecularly imprinted polypyrrole modified pencil graphite electrode for CPF. The MIP film was prepared by in situ electropolymerization of pyrrole on the PGE surface in the presence of CPF. The interaction between CPF and CPF-imprinted cavities on the PPy layer was monitored by EIS without a label, and the obtained LOD was 1 × 10^−8^ mol L^−1^. The proposed impedimetric sensor was successfully applied to CPF-added water, leaf, and soil samples. This strategy is a simple label-free but is not suitable for trace chlorpyrifos analysis as the previously reported strategy using MIP-Aptamer [93].

A MIP for triazophos (TAPs), another organothiophosphate insecticide was developed by Li et al. [94]. *O*-hydroxyphenol was electropolymerized on an AuNP-carbon nanotube modified GCE (MIP-AuNP-CNT/GCE) in the presence of template TAP. The electrochemical response of TAP at the TAP-imprinted polyhydroxyphenol modified AuNP-CNT/GCE was investigated by direct CV detection and a LOD of 9 × 10^−8^ mol L^−1^ was achieved; the sensor was successfully applied to monitor TAPs in vegetable samples. Using pyrrole as a functional monomer, Capoferri et al. [95] proposed a low-cost, simple, and easy MIP for dimethoate (an organophosphate insecticide). A MIP film was synthesized by electropolymerization of pyrrole in the presence of dimethoate as a template molecule on the surface of a GCE using CV. Being dimethoate electro-inactive, K_3_[Fe(CN)_6_] was used as a probe for the indirect quantification of the analyte via the decrease of redox peaks observed upon binding of the target analyte. Detection of dimethoate at a low nanomolar range was achieved. In this work, the MIP sensor was coupled with a microextraction by packed sorbent strategy for the selective and sensitive detection of dimethoate in wheat flour samples.

Li et al. [96] proposed a supramolecular imprinted sensor for carbofuran (carbamate pesticide) detection. The sensor was based on a functionalized MWCNT-supported Pd-Ir composite and methylene blue as a catalyst. A GCE which was doubly modified with MWCNT/Pd-Ir and the methylene blue doped MIP was used to prepare a sensor for carbofuran detection. First, 4-tert-butylcalix [8] arene (4TB[8]A)-CBF (4TB[8]A-CBF) supramolecular inclusions were prepared via self-assembly. Then, the MB-doped MIP was prepared via electropolymerization, using MB-doped o-phenylenediamine as a functional monomer and the 4TB[8]A-CBF supramolecular inclusions as the template molecules. Due to the electroactivity of carbofuran, it was designed as a switch to control the current intensity by its elution and adsorption in the MIP. Glyphosate is the most commonly used herbicide in the world. It has been reported that glyphosate is a non-electroactive compound that cannot be measured at accessible potentials. Xu et al. [97] designed a molecularly imprinted PPy for sensitive voltammetric determination of glyphosate. A composite of urchin-like AuNPs and Prussian Blue was electrodeposited on an indium/tin oxide (ITO) glass to serve as a low-potential redox mediator and electron transfer carrier. Then, PPy was imprinted with glyphosate to form a MIP on the surface of the ITO electrode through hydrogen bonds between glyphosate and pyrrole. The LOD was 5 × 10^−10^ mol L^−1^. Do et al. [98] proposed an electrochemical sensor for the sensitive and selective detection of glyphosate based on a molecularly imprinted metal-organic framework (MOF). The sensor was prepared through electropolymerization of p amino thiophenol functionalized AuNPs in the presence of glyphosate as a template molecule. The extraction of the template leads to the formation of cavities that can specifically recognize and bind glyphosate through hydrogen bonds between glyphosate molecules and aniline moieties. The performance of the developed sensor for the detection of glyphosate was investigated by linear sweep voltammetry using a ferrocyanide-ferricyanide solution as a redox probe. The molecularly imprinted sensor exhibited a broad linear range, between 5 × 10^–15^ to 5.9 × 10^–9^ mol L^−1^ and a quantification limit of 5 × 10^−15^ mol L^−1^. The developed sensor was successfully applied to detect glyphosate in tap water samples. In another context, Mazouz et al. [99] also proposed MIP-based gravimetric and electrochemical sensors for picomolar detection of glyphosate herbicide.

2,4-dichlorophenoxyacetic acid (DPA) has been listed as the drinking water contaminant with a critical level of 0.07 mg L^−1^ by the Environmental Protection Agency (EPA). The detection of DPA at low concentrations is a significant challenge. Recently, Wang et al. [100] developed Dendrimer-like amino-functionalized hierarchical porous silica nanoparticles (HPSNs-NH_2_) as a host material for the imprinting and sensing of DPA. The HPSNs-NH_2_ nanoparticles were synthesized using an emulsion method. The selective MIP was prepared on the HPSNs-NH_2_-modified electrode via electropolymerization using DPA as a template and o-phenylenediamine (OPD) as a monomer. The porous structure of HPSNs-NH_2_ reduced the diffusion limitations of the analytes, enhanced the accessibility, and increased the surface area of the sensor, while the MIP layer offered the ability to recognize and quantify the target DPA by using ferrocyanide-ferricyanide as a redox probe. The detection limit was down to 1 × 10^–11^ mol L^−1^ using DPV. This method has been applied to detect DPA in bean sprout samples with good recoveries.

#### 5.2.2. Veterinary Drugs

The combination of MIPs selectivity and high sensitivity of the electrocatalytic reaction of bioactive enzymes for signal amplification is a novel strategy proposed by Que et al. [101] for the sensitive detection of streptomycin (STR) in food. The MIP was fabricated via co-polymerization of o-phenylenediamine and aniline on a gold substrate in the presence of the template STR. The assay was based on the competitive binding of free STR and glucose oxidase-labeled STR (GOx-STR). The enzymatic catalysis of glucose produces hydrogen peroxide detected by differential pulse voltammetry, with a LOD of 1 × 10^−16^ mol L^−1^ STR. The system was further validated and evaluated with STR-spiked samples, including honey and milk. The recovery was between 82% and 124%. This work aims to set up a new class of molecularly imprinted copolymer sensing strategies with sensitivity enhancement using the enzyme catalytic properties. Another research group applied the same competitive strategy. Liu et al. [102] reported a MagMIP based on nanogold-encapsulated poly(o-phenylenediamine) shell on magnetic iron oxide cores and used a direct competitive type assay format for the electrochemical determination of Streptomycin (STR) residues. The MagMIP nanospheres were synthesized by using Au (III)-promoted molecularly imprinted polymerization with STR templates and o-phenylenediamine as a monomer on magnetic beads. The assay is carried out with a competitive-type assay mode between target molecules and glucose oxidase-labeled streptomycin for molecular imprints on the magnetic beads. The signal is generated and amplified based on the catalytic oxidation of labeled glucose oxidase toward glucose in the detection solution with the help of redox-active MagMIP nanospheres. SWV at an ITO electrode was employed to monitor the electrochemical reaction. The sensor provided good analytical characteristics (LOD = 2 × 10^−14^ mol L^−1^) and demonstrated successful applicability to the analysis of spiked milk and honey samples. This work provides a proof of concept strategy based on magneto-controlled MIP- electrochemical sensor coupled competitive type assay for simple and sensitive determination of small molecules.

Moro et al. [103] proposed a direct electrochemical detection method for β-lactam antibiotics using cefquinome (CFQ) coupled to MIP. The MIP-CFQ was constructed at MWCNTs modified graphite screen-printed electrodes (MIP-MWCNTs-Gr/SPEs) by electropolymerization in 0.1 M sulfuric acid with a monomer(4-aminobenzoic acid): template (CFQ) ratio of 5:1, for the creation of a sensor for β-lactam antibiotics detection in milk. A LOD of 5 × 10^−8^ mol L^−1^ by SWV at the modified sensor was obtained.

Lian et al. [104] developed a novel low-cost, stable and sensitive electrochemical sensor based on MIP and chitosan-silver nanoparticles (CS-SNP)/graphene-MWCNTs (GR-MWCNTs) modified gold electrode for the detection of neomycin (aminoglycoside antibiotic produced by *Streptomyces fradiae*). MIPs were synthesized by electropolymerization using neomycin as a template and pyrrole as a monomer. Direct amperometry detection was used, demonstrating a LOD of 8 × 10^−9^ mol L^−1^.

Chloramphenicol antibiotic has been applied in veterinary medicine and in humans to treat Gram-positive and Gram-negative infections. It should be mentioned that the residues of CAP have serious side effects on public health [69]. Zhao et al. [105] developed an MIP electrochemical sensor based on platinum thin-film microelectrode (Pt TFME) for the detection of chloramphenicol in food samples. The MIP sensor was synthesized by electropolymerization of o-phenylenediamine (OPD) in the presence of CAP on the surface of Pt TFME. Indirect SWV was used for electrochemical detection with K_3_Fe (CN)_6_ as an electroactive probe. A detection limit of 4 × 10^−10^ mol L^−1^ in honey and milk samples was achieved. Recently, Roushani et al. [106] designed an ultrasensitive impedimetric biosensor based on MIP and DNA aptamer of 41 bases for the detection of chloramphenicol. The biosensor was composed of aptamer-MIP/silver nanoparticles (AgNPs)/3 aminomethyl pyridine functionalized graphene oxide (3-ampy-rGO)/GCE. 3 aminomethyl pyridine functionalized graphene oxide (3-ampy-rGO) was applied to modify the GCE. After the modification of GCE, the AgNPs were immobilized on the NH_2_ groups of the 3-ampy-rGO via the formation of Ag-N bonds. The aptamer was charged to the electrode surface via the interaction between the AgNPs and the -NH_2_ group of the aptamer. Subsequently, the resorcinol (monomer) was electropolymerized around the aptamer/CAP complex. The MIP, in this sensor, acted as a second recognition element in synergy with the incorporated aptamer. It is interesting to note that the exact dual property of the molecular imprinting polymers and the aptamers led to excellent detection properties. The chloramphenicol was measured indirectly using hexacyanoferrate as an electrochemical redox by EIS. A detection limit of 3 × 10^−13^ mol L^−1^ was obtained. This strategy was successfully applied for the determination of Chloramphenicol in a real complex sample such as milk.

Wen et al. [107], proposed a nanowell-based molecularly imprinted electrochemical sensor for sensitive and selective detection of 17β-estradiol in food samples. A nanowell gold film with a thickness of 120 nm and pore size of ∼20 nm was immobilized onto a gold electrode surface to form a nanowell-based electrode. MIPs was then electropolymerized onto the nanowell-based electrode using 4-aminothiophenol as a monomer and 17β-estradiol as a template. This sensor surface exhibited a 3D-nanowell structure with a high surface area. Its enhanced electron-transport ability, while MIPs afford stronger recognition capability with higher selectivity and specificity and a lower detection limit of 1 × 10^−13^ mol L^−1^ using direct CV as electrochemical technique. Despite CV is known as low sensitive electrochemical technique (used as a characterization technique) because of its capacitive current, in this work, LOD is low. This demonstrates the high conductivity and electron transfer of the nanowell gold used for the modification of the electrochemical sensor. Wang et al. [108] developed a Molecularly imprinted electrochemical sensor to determine olaquindox (OLA) (growth-promoting and antibacterial agent), using AuNPs with MIP (AuNPs@MIP) and carboxylated MWCNTs (cMWCNTs). A GCE was modified with cMWCNTs and further with electrodeposited AuNPs using chronoamperometry. Then, the formation of MIP on the MIP-AuNPs-cMWCNTs/GCE was carried out via electropolymerization using OLA as a template and o-PD as a monomer. The developed MIP sensor provided a LOD of 3 × 10^−8^ mol L^−1^ for OLA determination in food and feedstuffs samples by direct DPV measurement.

The interactions between the template molecule and the functional monomers are the key to the successful imprinting process. This selection of the suitable functional monomer can be carried out using 1H-NMR titration and a computational calculation for further investigation of the template-monomer interactions. Recently, Dechtrirat et al. [109] developed a sensitive electrochemical MIP sensor for salbutamol (growth-promoting agent) detection based on a graphene/PEDOT: PSS modified SPCE using computational calculations prior to MIP synthesis. A suitable functional monomer was selected from several electropolymerisable monomers that can form multiple non-covalent bonds with salbutamol (template molecule), including 3-aminophenyl boronic acid resorcinol, o-phenylenediamine, and scopoletin. Within this study, The MIP layer was constructed on top of the conductive nanocomposite graphene/PEDOT: PSS by co-electropolymerization of 3-aminophenyl boronic acid and o-phenylenediamine as best functional monomers in the presence of salbutamol. Salbutamol was determined in real swine meat and feed samples with a good detection limit of 1 × 10^−10^ mol L^−1^ using DPV.

Sulfonamides in food, environmental water, and feed are a major concern for both aquatic ecosystems and public health because they may lead to the health risk of drug resistance [122]. Recently, Turco et al. [110] developed an amperometric sensor for sulfamethoxazole based on molecularly imprinted polydopamine on a gold electrode surface. A double-step procedure was used for sensor fabrication consisting of molecularly imprinted polydopamine (PDA-MIP) electropolymerization on a gold electrode, and template removal through a simple washing procedure with diluted acetic acid. SMX detection was then performed amperometrically. The MIP sensor evidenced a linear range from 8 × 10^−7^ to 2 × 10^−4^ mol L^−1^ and a good selectivity in the presence of other structurally related molecules such as sulfadimethoxine in spiked milk samples. Wei et al. [111] synthesized MIPs/NiCo_2_O_4_ nanoneedle arrays on a 3D graphene electrode for the determination of sulfadimidine (SM) residue in food. Firstly, NiCo_2_O_4_ nanoneedle arrays were decorated on free-standing and highly conductive 3D graphene by a hydrothermal process. Then, a 3D MIP sensor was prepared by electropolymerizing of pyrrole onto NiCo_2_O_4_ nanoneedle arrays in the presence of SM as a template molecule. The synergistic effect of mesoporous NiCo_2_O_4_ nanoneedle arrays, 3D graphene, and MIP were conducive to obtaining desirable sensitivity, linear range, and LOD of 6 × 10^−13^ mol L^−1^. Sun et al. [112] developed a molecularly imprinted electrochemical sensor with CuS micro flowers as an electron transfer probe and Au@COF for signal amplification for the determination of sulfathiazole (STZ). The selectivity of the MIP (imprinted with STZ) was coupled with the high electrical conductivity of an AuNP-decorated covalent organic framework (Au@COF) for enhancing the electrochemical signal. The proposed sensor had a LOD of 4 × 10^−12^ mol L^−1^, and chicken liver and pork liver were used as real samples.

#### 5.2.3. Process and Package Food Contaminants

Graphene has attracted increasing attention due to its unique nanostructure and properties. Large surface area, fast electron transfer rate, excellent electrical conductivity allow graphene to be used as a sensing element in electrochemical detections. Tan et al. [113] designed an electrochemical sensor based on molecularly imprinted PPy/graphene quantum dots (MIP-Py-GQDs) composite for the detection of BPA in water samples. A MIPPy/GQDs composite layer was prepared by the electropolymerizing of pyrrole on a GCE with BPA as a template. Indirect detection of BPA by the decrease of peak currents of K_3_[Fe (CN)_6_] at the MIP-Py-GQDs electrode using CV and differential pulse voltammetry (DPV) led to a LOD of 4 × 10^−8^ mol L^−1^. The sensor was applied for the detection of BPA in tap and seawater samples. Recently, Tutku Beduk et al. [114] designed a one-step electrosynthesized MIP on laser scribed graphene (LSG) electrode for bisphenol A. Electrochemical measurements were carried out directly using DPV. The straightforward and fast fabrication of LSG electrodes by CO_2_ laser provides an easy and mask-free mass production of the flexible three-electrode system. The electro-polymerization of pyrrole onto the LSG sensor allowed an easy formation of a biorecognition layer, leading to a susceptible and selective electrochemical device. The proposed sensor exhibited high selectivity towards BPA compared to its structural analogs and good reusability. The engineered sensor has been successfully used for the detection of BPA in tap, mineral water, and plastic samples with a low LOD of 8 × 10^−9^ mol L^−1^.

#### 5.2.4. Mycotoxins

Mycotoxins or fungal contaminants are also major biological hazards, while few research papers on MIP-based electrochemical sensors for mycotoxins detection have been reported. The development of an accurate analytical method for the detection of mycotoxins is highly needed to satisfy food safety requirements [123,124]. Recently, Huang et al. [115] applied thionine as both functional monomer and signal indicator to form an electrochemical sensor for the detection of patulin that is produced by *Aspergillus*, *Penicillium*, and *Byssochlamys* species. By electropolymerizing, molecularly imprinted polythionine was synthesized on the Pt nanoparticles (PtNPs)-nitrogen-doped graphene-modified GCE. With two amino groups, thionine can not only form poly (thionine) film but also drives patulin targets into the MIP through a hydrogen bond. Though functional monomer has good electron transferability, the combination of patulin and MIPs could decrease the electric conductivity of MIP film, decreasing the reduction peak current. Therefore, this sensor displayed an excellent performance for patulin detection over the range of 1 × 10^−14^ to 1 × 10^−11^ mol L^−1^ with a detection limit of 6 × 10^−15^ mol L^−1^, and successfully detected patulin in real apple and grape juice samples using DPV as an electrochemical technique. Pacheco et al. [116] designed a simple electrochemical sensor for ochratoxin A (OTA) detection through the modification of a GCE with MWCNTs and MIP. The MWCNTs dramatically promoted the sensitivity of the developed sensor, while PPy imprinted with OTA served as the selective recognition element. Pyrrole was selected as a monomer for the formation of the MIP because it can form hydrogen bonds between the oxygen groups of OTA and the N–H group of PPy. The imprinted PPy film was prepared by electropolymerizing of pyrrole in the presence of OTA as a template molecule via CV. The electrochemical oxidation of OTA at the developed sensor was investigated by CV and DPV. The developed MIP-MWCNT/GCE sensor showed a LOD of 4 × 10^−9^ mol L^−1^. The MIP-MWCNT/GCE sensor was simple to fabricate and easy to use and was successfully applied to the determination of OTA in spiked beer and wine samples, with recoveries between 84 and 104%, without the need of a sample pre-treatment step. Hu et al. [117] synthesized an ionic liquid-assisted self-assembly molecular imprinting strategy for Zearalenone (F-2 toxin produced by Gibberella species and Fusarium graminearum) based on ionic liquid functionalized boron-doped ordered mesoporous carbon AuNPs composite (BOMC-IL-Au NPs). During the composite synthesis, well-dispersed and uniform AuNPs are deposited on the surface of IL-modified BOMC due to the strong electrostatic interaction between AuCl_4_ and positively charged IL. For molecular imprinting, the BOMC-IL-Au NPs/GCE is immersed into a p-amino thiophenol (*p*-ATP) solution and template solution in turn. Thus, the mercapto group contained p-ATP self-assembles on the AuNPs. Subsequently, the template molecules self-assemble onto the composite to form a dense template layer, because of the hydrophobic interaction, π-π, and hydrogen bond between the template and IL/or p-ATP. After electro-polymerization, the template layer is embedded into the p-ATP polymer membrane and produces lots of imprinted sites. Hence, the obtained sensor exhibited high sensitivity and selectivity with a detection limit of 3 × 10^−16^ mol L^−1^, using [Fe(CN)_6_]^3−/4−^ probe and SWV.

Deoxynivalenols (DON) are trichothecene mycotoxins produced by various species of *Fusarium graminearum* in wheat, barley, maize, and cereal-based food products. An impedimetric sensor based on a MIP prepared on the surface of an (AuSPE) for the analysis of deoxynivalenols has been developed by Radi et al. [118]. Under optimal conditions, the charge transfer resistance (Rct) showed a linear relationship with the logarithm of the DON concentration in the range of 2 × 10^−11^ to 2 × 10^−9^ mol L^−1^, with a detection limit of 1 × 10^−12^ mol L^−1^. The developed MIP sensor can be recycled at least 30 times without loss of affinity to the template molecule. The sensor has been employed successfully for the determination of DON in commercial cornflake food samples. Citrinin (CIT) is another mycotoxin produced by *Penicillium citrinum*. It is a mold that can be present in many foods (e.g., cheese, red yeast rice, etc.). CIT is mutagenic and resistant to decomposition. Owing to these effects, CIT is one of the most dangerous chemicals, according to the World Health Organization [119]. An imprinted electrochemical surface based on GCE modified with palladium nanoparticles (PdNPs) functionalized graphene quantum dots (GQDs) was proposed for CIT analysis by Akyıldırım et al. [119]. CIT imprinted electrochemical surface was shaped in the presence of pyrrole as monomer and CIT as a template. The nano sensor showed a LOD of 2 × 10^−10^ mol L^−1^ using DPV, demonstrating good stability (up to 60 days). Besides, the prepared electrode was applied to chicken egg samples for CIT detection with high selectivity and satisfactory recoveries in the chicken egg matrix in the presence of ochratoxin A and ochratoxin B.

The use of dummy templates to prepare MIP has several advantages: reducing toxic exposure during polymer synthesis and addressing leaching templates from the imprinted polymer. Dummy template MIP strategy is widely used for MIP mycotoxin for sample preparation coupled with mass spectrometry analysis [125]. However, up to date, this strategy is not explored yet for the electrochemical sensors based on MIP to detect mycotoxin in food samples.

#### 5.2.5. Bacterial Contaminants

Compared with veterinary drugs and pesticides, the number of publications on the analysis of bacterial contaminants is much lower.

Idil et al. [120] developed a capacitive sensor based on MIP for the detection of Escherichia coli. The *Escherichia coli* cells were first immobilized on the surface of a glass slide. Then, the templates were contacted with *N*-methacryloyl-l-histidine methyl ester (MAH) and 2-Hydroxyethyl methacrylate (HEMA) as monomers and ethylene glycol dimethacrylate (EGDMA) as a cross-linking. After UV photo-polymerization and removal of bacterial stamp, this sensor achieved a LOD of 70 CFU mL^−1^ using indirect electrochemical detection by CV in river water and apple juice as real samples. There have been only a few reports focusing on whole-cell imprinting, so this study provides an excellent model for the detection of pathogenic and spoilage bacteria.

Label-free biosensors can provide rapid bacterial detection that is needed in many areas, including food safety, clinical diagnostics, biosecurity, and biosafety. Whole-cell imprinted polymers can be used as recognition elements in biosensors for the selective detection of bacteria. 3-aminophenyl boronic acid has been used for the electrochemical manufacture of a cell imprinted polymer (CIP). The use of a monomer with a boronic acid group, with its ability to interact specifically with a cis diol, has enabled the formation of a polymer network with both morphological and chemical recognition abilities. The attractive feature of the envisaged approach is the reversibility of the *cis*-diol-boronic group complex, which facilitates the fabrication of the captured bacterial cells and the subsequent regeneration of the CIP. *Staphylococcus epidermidis* was used as a model target bacterium for CIP, and electrochemical impedance spectroscopy (EIS) was used for the label-free detection of target bacteria. The modified electrodes showed a linear response in the range of 10^3^–10^7^ CFU mL^−1^. A selectivity study also showed that CIP can distinguish its target from non-target bacteria of similar shape. CIPs have a high affinity and specificity for bacterial detection and provide a switchable interface that allows the bacterial cell to be easily removed by competitive displacement with fructose and deionized water washout [121].

## 6. Application of MIPs in Sample Preparation for Food Safety Analysis

Selectivity is probably the most important goal in analytical food chemistry. It becomes imperative in the case of trace analysis of target species in real matrices, which are often quite complex, such as environmental, biological, and food samples. Ideally, the analytical method should be accurate, reproducible, and highly selective (or specific) toward the target analyte. In particular, selectivity should be pursued both in the instrumental detection technique and/or in the sample preparation step. Since the latter is strictly connected with potential matrix effects occurring in the final quantification, with the overall method sensitivity, selectivity is essential in the sample pretreatment. Depending on the matrix, sample preparation is necessary for the extraction/enrichment/clean-up of trace compounds. The most used pre-concentration/clean-up technique is solid-phase extraction (SPE) which has almost totally replaced the traditional liquid-liquid extraction (LLE). Besides, different ways of making SPE have been developed, such as dispersive SPE (d-SPE), magnetic SPE (MSPE), stir-bar sorptive extraction (SBSE), and solid-phase microextraction (SPME).

**Figure 5:**
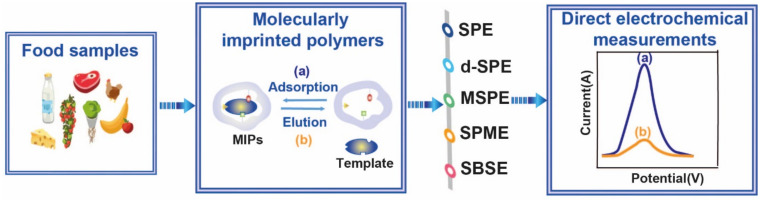
Application of MIP in sample preparation for the electrochemical analysis of food. SPE: Solid-phase extraction, *d*-SPE: Dispersive SPE, M-SPE: Magnetic SPE, SPME: Stir-bar sorptive extraction, SBSE: Solid-phase microextraction.

MIPs are one of the major sorbents used in SPE (SPE, d-SPE, MSPE, SPME, and SBSE procedures) [126] for selective determination of residual pesticides, veterinary drugs, mycotoxins, and persistent organic pollutants in food matrices [127]. Figure 5 illustrates the combination process of MIP-based SPE and electrochemical detection for food samples.

**Figure 6:**
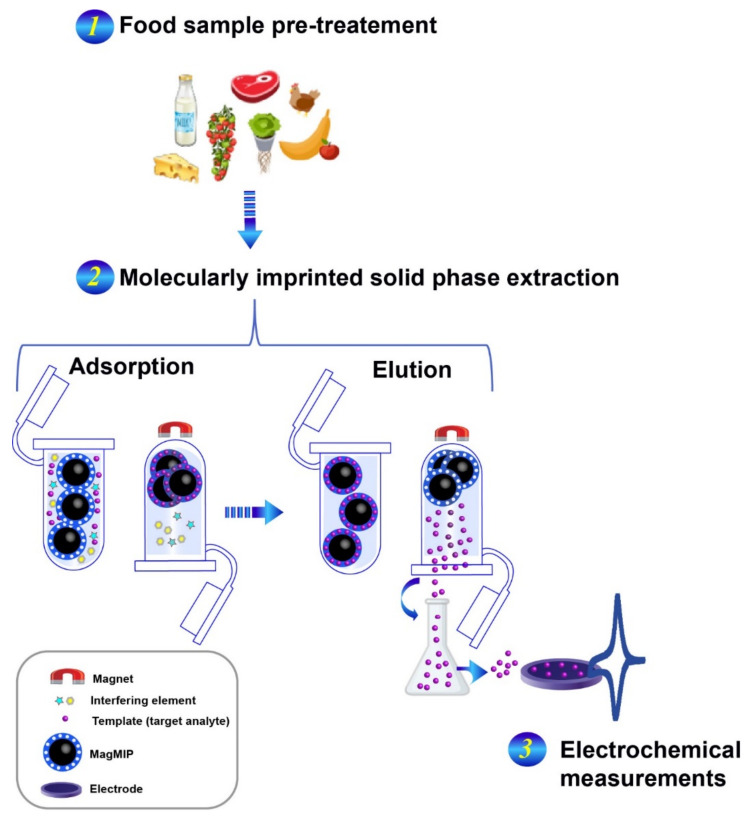
Schematic procedure for the electrochemical sensing of the target analyte coupled magnetic-MIP as solid-phase extraction from a complex food sample.

M-SPE has received significant attention recently in food clean-up because it can make the purification and separation processes faster and more accessible owing to the non-use of centrifugation or filtration. The MagMIPs can be concentrated easily with an external magnet onto the surface of a transducer (Figure 6), improving the sensor’s sensitivity.

Figure 6 shows a graphical sketch of the electrochemical sensing of the target analyte coupled magnetic-MIP as solid-phase extraction.

In general, the combination of sample preparation using MIPs and electrochemical detection in the field of food is poorly reported and discussed in the literature. Table 3 lists the literature data about the use of MIPs in sample preparation in the field of food analysis combined with electrochemical sensors. The most important parameters related to MIP preparation and the electrochemical measurement were provided for each target analyte in this table.

For the detection of fenbendazole (FBZ), an anti-parasitic widely used in veterinary medicine against endoparasites in food-producing animals, a MIP-based SPE of FBZ in the beef liver as a complex food sample coupled to SWV at carbon-fiber microelectrodes sensor was designed by Prada et al. [130]. A non-covalent imprinting synthesis strategy using a 1:8:22 ratio of template, MAA, and ethylene glycol dimethacrylate at 62 °C for 16 h was used. The bulk-polymerized MIPs were coupled to an SPE method (MISPE). A detection limit of 2 × 10^−7^ mol L^−1^ with a good recovery above 95% using SWV was reported.

Li et al. [135] developed a tyramine detection system that integrated sample preparation using SPE and detection by an electrochemical sensor MIP effectively reduced interfering signals in the subsequent electrochemical detection. MAA, trimethylolpropane trimethacrylate (TRIM), acetonitrile, and AIBN were used as the monomer, crosslinker, porogen solvent, and initiator, respectively. The polymerization was carried out at 60 °C for 24 h. The electrochemical sensor platform was based on a conductive polymer polystyrene sulfonate (PEDOT: PSS) on an SPCE, followed by electrodeposition of AuNPs (SPCE/PEDOT: PSS/AuNPs), and further signal amplification was achieved by fixing 1-methyl-4-mercaptopyridine (1-m-4-MP) to AuNP on working electrodes through gold–sulfur bonds, for the selective detection of tyramine. A linear concentration range (5 × 10^−9^–1 × 10^−7^ mol L^−1^) with LOD of 2 × 10^−9^ mol L^−1^ was obtained by analyzing tyramine in spiked serum and milk.

Zhang et al. [132] developed a MIP sensor combined with magnetic molecularly imprinted SPE (MMISPE) for the determination of dibutyl phthalate (DBP) in soybean milk and milk samples. The MagMIPs (MIP/nano-Fe_3_O_4_/SiO_2_) were synthesized as sorbent in SPE to extract DBP and as a sensing element to improve the selectivity of the imprinted sensor. The MIP was prepared by using DBP, MAA, EGDMA, and AIBN as a template, monomer, crosslinker, and initiator, respectively. The polymerization has occurred at 60 °C for 12 h. An indirect detection method using hexacyanoferrate and DPV was developed. The linear range was from 4 × 10^−11^ to 4 × 10^−6^ mol L^−1^ and the LOD 2 × 10^−13^ mol L^−1^ demonstrating high sensitivity toward the selective detection of DBP in complex milk samples with satisfactory recoveries of 96–100%.

Fast synthesis of MagMIPs was proposed by Messaoud et al. [36], for the selective extraction and electrochemical determination of Bisphenol A (BPA) in tap and mineral water samples. The MagMIPs were synthesized by ultrasound bath using free radical polymerization for 2 h. The resulting material exhibited high selectivity towards BPA. The developed Mag-MIP was combined with a voltammetric sensing strategy based on MIP-AuNPs-Carbon black/SPE to detect BPA in water with a LOD of 9 × 10^−9^ mol L^−1^; this Mag-SPE is a promising alternative to traditional sample pretreatment using classical cartridges prior to analytical detection and can be used for monitoring water safety.

Hassan et al. [134] proposed the use of MagMIPs for the preconcentration of histamine in fish samples, followed by direct electrochemical readout of this biogenic amine from the surface of magneto-actuated electrodes. These MagMIPs were synthesized by the core-shell method using histamine as a template and 2-vinyl pyridine as a functional monomer. The analytical performance was validated to determine histamine in scombrid fish samples with recovery values ranging from 97 to 102% with a LOD of 2 × 10^−6^ mg L^−1^ which is below the fish spoilage index (50 mg kg^−1^) according to legislation. The same research group proposed the same strategy to detect Methyl parathion in tuna and catfish [129], 1-chloro-2,4-dini-trobenzene (CDNB) as a toxic xenobiotic compound known to cause oxidative stress cell death and allergic reactions were also preconcentrated using magneto-actuated electrodes from the river, and tap water by Ruiz-Córdova et al. [136].

Dummy MIP for dichlorodiphenyltrichloroethane (DDT) detection was proposed by Miao et al. [128]. BPA and dopamine were used as a virtual-template molecule and functional monomer, respectively. The MagMIPs were used for specific adsorption and efficient extraction of target molecules 4,4’-DDT from food samples. The developed impedimetric sensor based on MagMIPs showed a good linear range, 1 × 10^−11^ to 1 × 10^−3^ mol L^−1^ with a LOD of 6 × 10^−12^ mol L^−1^. Despite the important analytical results obtained in this work, the use of dopamine as a functional monomer to prepare MIP still faces fundamental challenges [137].

Green synthesis of biocompatible MagMIPs was recently performed using tetracycline as a template and Zein (a class of prolamin protein found in maize) as cross-linker. This study proved that the produced MagMIPs were ideal adsorbents for the selective extraction and enrichment of tetracycline. The developed SPE method was successfully combined with a portable electrochemical voltammetric sensor. The proposed strategy allows simple sample collection combined with a portable electrochemical workstation to monitor tetracycline in milk. The obtained results showed that this analytical procedure could be applied to selectively detect tetracycline in milk and other foods with a LOD of 6 × 10^−11^mol L^−1^ [131]. A similar approach was successfully used to detect curcumin by the same research group [133].

## 7. Conclusions and Future Perspectives

This review critically discusses the recent progress in MIP-based electrochemical sensors for food safety. A brief overview of MIPs and electrochemical sensors has been reported, followed by a presentation of the recent achievements for various MIPs-based electrochemical sensors highlighting the main achievements obtained. Undoubtedly the number of papers in this field indicates that there is a large scientific interest by researchers worldwide. This can be justified by the necessity to find sensitive and rapid screening tests coupled with the required selectivity. In this respect, electroanalytical tools coupled with MIPs really represent a fascinating solution. The performance of every single analytical approach is difficult to compare since the target and the type of polymerization strongly influence the selectivity and the sensitivity of the methods. However, the very low detection limits obtainable, the good reproducibility, as well as the good recovery reported for some targets, make the approach feasible for the realization of screening assays for food safety. Moreover, some MIPs improvements have translated the concept of reusability also for MIPs chemically synthesized as for the MagMIPs, allowing reliable, economical, and sustainable applications. On the other hand, modern protocols regarding electropolymerization of MIPs allowing to obtain extremely reproducible MIPs onto different supports also taking advantage of different nanomaterials.

However, the majority of papers do not report the validation of the methods that are mandatory for the application of the assays in reality. Moreover, sometimes the preparation of the MIPs, particularly for chemical synthesis, takes a long time, and data on reproducibility using different batches are not clearly reported. Despite this, the use of MIPs coupled with electroanalytical tools it will certainly give rise to future evolutions, in particular regarding integrated devices realized with sustainable materials and substrates. For example, the introduction of new polymerization techniques able to synthesize MIPs in thin films onto nanomaterials will lead to the design of new efficient sorbents for different food analytes. The use of microfluidics implemented onto low-cost electrochemical platforms can lead to the development of multiplexed approaches if coupled with the appropriate MIP extraction strategy, providing miniaturization, low reagent consumption, and automation of point-of-need/care systems. The application of MagMIPs in food analysis, in our opinion, will continue to expand at a faster rate than ever before. They represent the analog of immunomagnetic beads that are very well spread in the development of immunoassays. Studies based on MagMIPs should be focused on the performance of the extraction and separation procedure proposing new configurations for the synthesis of magnetic sorbents with improved selectivity and yield of extractions. As a final consideration on targets, the majority of the electrochemical-MIP reported are focused on small size targets, we are sure that in the future considering, the soft polymerization or epitope imprinting approaches that many different researchers are applying for proteins and bacteria different electrochemical-MIPs for proteins (i.e., allergens) or pathogens will be prepared and will be an effective alternative to classical immunoassays for these targets.

## Figures and Tables

**Table 1:** Chemically synthesized MIPs-combined electrochemical sensors for food contaminants detection.

Category	Analyte/Template	Sensing Scheme/Configuration	Monomer/Crosslinker/Extraction Solution	Type of Measurement	Detection Method	LR and LOD	Real Sample	Ref.
**Pesticides**	**Methyl parathion**	MIP–IL–Gr/GCE	/MAA /EGDMA /ethanol (with the aid of ultrasonication)	Direct	DPV	1 × 10^−8^ to 7 × 10^−6^ mol L^−1^; 6 × 10^−9^ mol L^−1^	Cabbage Apple peel	[53]
	**Acephate and trichlorfon**	MIP-Fe_3_O_4_-MWNTs COOH-CS/GCE	APTES /TEOS /Methanol-acetic acid solution (9:1, *v*/*v*)	Direct	DPV	1 × 10^−4^ to 1 × 10^−10^ mol L^−1^ for acephate and 1 × 10^−5^ to 1 × 10^−11^ mol L^−1^ for trichlorfon; 7 × 10^−11^ mol L^−1^ for acephate and 9 × 10^−12^ mol L^−1^ for trichlorfon	Kidney bean and cucumber samples	[54]
	**Methyl parathion**	MIP/CPE	MAA /Divinylbenzene /Methanol-acetic acid solution (9:1, *v*/*v*)	Indirect	CV	1 × 10^−12^ to 8 × 10^−9^ mol L^−1^; 3.4 × 10^−13^ mol L^−1^	Soil and vegetables	[55]
	**Diazinon**	MIP/CPE	MAA /EGDMA /methanol	Direct	SWV	2 × 10^−9^ to 1 × 10^−7^ mol L^−1^; 8 × 10^−10^ mol L^−1^	Well water Apple fruit	[56]
	**Parathion**	MIP-beads/GCE	MAA /Divinyl benzene /Ethanol-water (*v*/*v* = 1:5)	Direct	SWV	1 × 10^−7^ to 1 × 10^−5^ mol L^−1^; 5 × 10^−8^ mol L^−1^	Pear juice	[57]
	**Malathion**	MIP –AU/SPE	Acrylamide /EGDMA /Methanol-acetic acid solution (9:1, *v*/*v*)	Indirect	DPV	3 × 10^−16^ to 3 × 10^−12^ mol L^−1^_;_ 2 × 10^−16^ mol L^−1^	Olive fruits and oils	[58]
	**Chlortoluron**	MIP-NiO/GCE	MAA /N, N’-(methylene)-bisacrylamide /ethanol	Indirect	Chronoamperometry	1 × 10^−8^ to 1 × 10^−4^ mol L^−1^; 2 × 10^−9^ mol L^−1^	Water samples	[59]
	**Chlorotoluron**	MIP -NiHCF/GCE	MAA /EGDMA /ethanol (25%)	Indirect	CV	5 × 10^−9^ to 1 × 10^−7^ mol L^−1^; 9 × 10^−10^ mol L^−1^	Water	[60]
	**Carbofuran**	MIP-rGO-Au/GCE	Methyl acrylic acid /EGMRA /Methanol-acetic acid solution (9:1, *v*/*v*)	Direct	DPV	5 × 10^−8^ to 2 × 10^−5^ mol L^−1^; 2 × 10^−8^ mol L^−1^	Cabbage Cucumber	[61]
	**Phoxim**	MIP-graphene/GCE	Acrylamide /EGMRA /acetic acid–methanol (3:7, *v*/*v*)	Direct	DPV	8 × 10^−7^ to 1 × 10^−4^ mol L^−1^; 2 × 10^−8^ mol L^−1^	Cucumber	[62]
	**Profenofos**	MIP -3D-CNTs/GCE	MAA /EGDMA/ Methanol-acetic acid solution (9:1, *v*/*v*)	Direct	AMP	1 × 10^−8^ to 2 × 10^−4^ mol L^−1^; 2 × 10^−9^ mol L^−1^	Vegetable samples	[63]
	**Imidacloprid**	MIP-rGO/GCE	P-vinylbenzoic acid /EGDMA /0.1 M phosphate-buffered solution (PBS, pH 7.2)	Direct	LSV	5 × 10^−7^ to 2 × 10^−5^ mol L^−1^; 1 × 10^−7^ mol L^−1^	Rice	[64]
	**Dinotefuran**	MIP-carboxylated PVC sensor	Acrylamide /EGDMA /Methanol-acetic acid solution (9:1, *v*/*v*)	Direct	Potentiometry	2 × 10^−6^ to 1 × 10^−2^ mol L^−1^; 2 × 10^−6^ mol L^−1^	Cucumber and soil samples	[65]
	**Cyanazine**	MIP/carbon paste electrode	Acrylamide /EGDMA /Methanol-acetic acid solution (9:1, *v*/*v*)	Direct	Cathodic stripping voltammetry	5 × 10^−9^ to 1 × 10^−6^ mol L^−1^; 3 × 10^−9^ mol L^−1^	Tomato, onion, lettuce, and rice	[66]
	**Hexazinone herbicide**	MIP/CPE	2-vinylpyridine /EGDMA /Methanol-acetic acid solution (9:1, *v*/*v*)	Direct	DPCSV	2 × 10^−11^ to 1 × 10^−10^ mol L^−1^; 3 × 10^−12^ mol L^−1^	Water	[67]
**Veterinary drugs**	**Ciprofloxacin**	MIP-Ch-Au/GCE	MAA /EGDMA Methanol-acetic acid solution (9:1, *v*/*v*)	Direct	DPV	1 × 10^−3^ to 1 × 10^−1^mol L^−1^; 2 × 10^−11^ mol L^−1^	Mineral and tap water samples, milk, and pharmaceutical tablets	[68]
	**Ampicillin**	MIP-Fe_3_N-Co_2_N/CC	NNDMA /EDMA /Methanol-acetic acid solution (9:1, *v*/*v*)	Indirect	DPV	6 × 10^−9^ to 9 × 10^−3^ mol L^−1^; 4 × 10^−10^ mol L^−1^	Milk	[69]
	**Kanamycin**	MIP-MWCNTs-Fe_3_O_4_/CE	MAA /EGDMA /Methanol/acetic acid (8:2, *v*/*v*)	Indirect	DPV	1 × 10^−10^ to 1 × 10^−6^ mol L^−1^; 2 × 10^−11^ mol L^−1^	Liver and milk	[70]
	**Cloxacillin**	MIP-GO -Au NPs/SPE	MAA /EGDMA /Methanol	Direct	DPV	1 × 10^−7^ to 8 × 10^−7^ mol L^−1^; 4 × 10^−8^ mol L^−1^	Milk	[71]
	**Toltrazuril**	MIPs-TiO_2_-rGO/Pt E	APTES /TEOS /HCl solution (0.5 M)	Indirect	DPV	1 × 10^−3^ to 1 × 10^−1^ mol L^−1^; 5 × 10^−4^ mol L^−1^	Chicken muscle and egg	[72]
	**Sulfanilamide**	MIP-GO/GCE	MAA /EGDMA Methanol-acetic acid solution (9:1, *v*/*v*)	Direct	SWV	6 × 10^−11^ to 6 × 10^−9^ mol L^−1^; 2 × 10^−9^ mol L^−1^	Milk	[73]
	**Sulfamethoxazole**	MIPs-H-GNs/GCE	MAA /EGDMA /Phosphoric acid aqueous solution (pH = 2.0).	Indirect	DPV	2 × 10^−11^ to 4 × 10^−9^ mol L^−1^; 5 × 10^−12^ mol L^−1^	Milk Honey Serum	[74]
	**Estradiol**	MIP-Fe_3_O_4_-GSH/Gold electrode	Aniline /0.5 mol L^−1^ HCl-alcohol	Direct	DPV	2 × 10^−9^ to 1 × 10^−7^ mol L^−1^; 3 × 10^−9^ mol L^−1^	Milk powder	[75]
	**Diethylstilbestrol**	MIP-AuNPs-MWCNTs-CS/GCE	Monomer: APTES Crosslinker: TEOS Extraction solution: methanol containing 0.5 mol/L HCl (*v*/*v* = 9:1)	Indirect	DPV	1 × 10^−10^ to 1 × 10^−6^ mol L^−1^; 2 × 10^−12^ mol L^−1^	Milk	[76]
**Process and package contaminants**	**Bisphenol A**	MIP–GR/ABPE	Chitosan /0.1 mol L^−1^ HCl	Direct	Derivative voltammetry	8 × 10^−8^ to 1 × 10^−5^ mol L^−1^; 6 × 10^−9^ mol L^−1^	Plastic bottled drinking water and canned beverages	[77]
		MIP sol-gel MWCNTs-Au NPs/Au Electrode	APTES /EGDMA /Methanol-acetic acid solution (9:1, *v*/*v*)	Direct	Amperometry	1 × 10^−7^ to 1 × 10^−3^ mol L^−1^; 4 × 10^−9^mol L^−1^	Honey and grape juice	[78]
		MIP-GO-APTES/GCE	APTES /EGDMA /Methanol-acetic acid solution (9:1, *v*/*v*)	Direct	DPV	6 × 10^−9^ to 2 × 10^−5^ mol L^−1^; 3 × 10^−9^ mol L^−1^	Mineral water and milk	[79]
		MIPs-Gr/ABPE	4-vinyl pyridine /EGDMA /methyl alcohol/acetic acid = 9:1,	Indirect	DPV	1 × 10^−12^ to 1 × 10^−3^ mol L^−1^; 3 × 10^−13^ mol L^−1^	Plastic pacifier, one-off plastic spoon, mineral water, and Water	[80]
		MIP/GCE	MAA /divinylbenzene /methanol /acetic acid (8/2, *v*/*v*)	Direct	Potentiometry	1 × 10^−7^ to 1 × 10^−6^ mol L^−1^; 6 × 10^−8^ mol L^−1^	spiked river watersamples	[81]
	**Diisononyl phthalate**	MIP/GCE	MAA /EGDMA /Methanol-acetic acid solution (9:1, *v*/*v*)	Direct	CV	5 × 10^−10^ to 1 × 10^−6^ mol L^−1^; 3 × 10^−10^ mol L^−1^.	Liquor	[82]
**Natural toxins**	**Tyramine**	MIP-MWCNT-AuNP-chitosan/GCE	Triethoxyphenylsilane silicic acid tetraethyl ester /EGDMA /Methanol-acetic acid solution (9:1, *v*/*v*)	Direct	AMP	1 × 10^−7^ to 1 × 10^−5^ mol L^−1^; 6 × 10^−8^ mol L^−1^	Yoghurt	[83]
	**Histamine**	MIP/Aluminum electrode	MAA /EGDMA /methanol (48 h), acetic acid/acetonitrile (1/1) (48 h) methanol (12 h)	Indirect	EIS	2 × 10^−9^ to 1 × 10^−8^ mol L^−1^; 2 × 10^−9^ mol L^−1^	No real sample just they varied the pH of the electrolyte from 5 to 12	[84]
**Bacterial contaminations or Biological hazards**	**N-acyl-homoserine-lactones**	MIP -Fe_3_O_4_-SiO_2_/CPE	MAA /EGDMA /Methanol-acetic acid solution (9:1, *v*/*v*)	Direct	DPV	2 × 10^−9^ mol L^−1^ to 1 × 10^−7^ mol L^−1^; 8 × 10^−10^ mol L^−1^	no data	[85]

Chitosan(CS), Nickel cobaltite (NiCo_2_O_4_), Iron Nitride- cobalt nitrides- nanowires array carbon cloth (Fe_3_N-Co_2_N/CC), Platinum nanoparticles (PtNP), Patinum electrode (PtE), Polyaniline (PANI), 2-Acrylamido-2-methyl-1propanesulfonic acid (AMPS), Graphene quantum dots (GQDs), Metal–organic frameworks(MOF), Dendrimer-like silica nanoparticles (HPSNs), Laser-scribed graphene (LSG), Magnetic actuation on glassy carbon electrode (*m*-GCE), O-phenylenediamine (OPD), Magnetic carbon paste electrode (MGCE), Graphene oxide(GO), Reduced Graphene Oxide (RGO), multi-walled carbon nanotubes(MWCNTs), Boron-doped diamond (BDD), magnetic nickel hexacyanoferrate (NiHCF), Iron(II) Nitride (Fe_3_N_2_), Ionic liquid–graphene composite film coated glassy carbon electrode (MIP–IL–Gr/GCE), Titanium dioxide (TiO_2_), Glutathione (GSH), Tin sulfide (SnS_2_), glassy carbon electrode (GCE), graphene quantum dots(GQDs), linear sweep voltammetry(LSV), polyaniline(PANI), Poly[2-methoxy-5-(3,7-dimethyloctyloxy)-1-4-phenylene (MDMO-PPV), 2-mercaptonicotinic acid (MNA), Polyoxometalates (POMs), 3-aminomethyl pyridine (3-Amp), 2-Mercaptobenzothiazole (MBT), 2-hydroxyethyl methacrylate (HEMA), PPV: Poly[2-methoxy-5-(3,7-dimethyloctyloxy)-1-4-phenylene vinylene.

**Table 2:** Electrosynthesized MIP-based sensors for food contaminants.

	Analyte/Template	Sensing Scheme/Configuration	Monomer/Extraction Solution	Type of Measurement	Electrochemical Technique	LR and LOD	Real Sample	Ref.
**Pesticides**	**Chlorpyrifos**	MIP/PGE	Pyrrole /HCl solution (pH 2.0)	Indirect	EIS	6 × 10^−8^ to 8 × 10^−7^ mol L^−1^; 1 × 10^−8^ mol L^−1^	Water, leaf, and soil	[92]
		MIP-aptamer-AuNR/GCE	Ortho-phenylenediamine and O-dihydroxybenzene/Methanol–nitric acid (4:1, *v*/*v*)	Indirect	DPV	1 × 10^−15^ to 4 × 10^−13^ mol L^−1^; 3 × 10^−16^ mol L^−1^	Apples Lettuces	[93]
	**Triazophos**	MIP-PHP-AuNPs-CNT/GC	*O*-hydroxyphenol/0.5 M H_2_SO_4_	Direct	CV	9 × 10^−8^ mol L^−1^	Celery Lettuce Spinage Cabbage	[94]
	**Dimethoate**	MIP/GCE	Pyrrole/ HCl solution pH 2	Indirect	SWV	1 × 10^−10^ mol L^−1^to 1 × 10^−9^ mol L^−1^; 1 × 10^−10^ mol L^−1^	Wheat flour	[95]
	**Carbofuran**	MIP-MWCNT-Pd-Ir- MB/GCE	O-phenylenediamine/Formic acid/ethanol solution (1:1, *v*/*v*)	Indirect	DPV	4 × 10^−11^ to 4 × 10^−9^ mol L^−1^; 2 × 10^−12^ mol L^−1^	Cowpea Chinese cabbage Tomato Apple	[96]
	**Glyphosate**	MIP-AuNPs-PB/ITO electrode	Pyrrole/Ethanol	Indiret	DPV	2 × 10^−9^ to 7 × 10^−9^ mol L^−1^; 5 × 10^−10^ mol L^−1^	Corn	[97]
		MIP-MOF/Gold electrode	*P*-aminothiophenol/PBS solution pH 7.2	Indirect	LSV	5 × 10^–15^ to 6 × 10^–9^ mol L^−1^; 4 × 10^–15^ mol L^−1^	Tap water	[98]
		MIP/Gold electrode	Pyrrole/Methanol + Acetic acid (1:1, *v*/*v*)	Indirect	SWV	1 × 10^−12^ to 1 × 10^−9^ mol L^−1^; 1 × 10^−12^ mol L^−1^	no data	[99]
	**2,4-dichlorophenoxyacetic acid**	MIP-HPSNs-NH_2_/GCE	OPD/Aqueous methanol 80%	Indirect	DPV	1 × 10^−10^ to 2 × 10^−8^ mol L^−1^; 1 × 10^–11^ mol L^−1^	Bean sprouts	[100]
**Veterinary drugs**	**Streptomycin**	MIP/Gold Electrode	Aniline and O-phenylenediamine/distilled water and ethanol	Direct	DPV	2 × 10^−14^ to 2 × 10^−11^ mol L^−1^; 1 × 10^−16^ mol L^−1^	Honey and milk	[101]
	**Streptomycin**	MIP-AU-STR-GrOX/ITO electrode	O-phenylenediamine/Ethanol (50%, *v*/*v*)	Direct	SWV	9 × 10^−14^ to 3 × 10^−11^ mol L^−1^; 2 × 10^−14^mol L^−1^	Honey and milk	[102]
	**Cefquinome**	MIP-MWCNTs-Gr/SPEs	4-aminobenzoic acid/0.1 M PB pH 12	Direct	SWV	5 × 10^−10^ to 1 × 10^−6^ mol L^−1^; 5 × 10^−10^ mol L^−1^	no data	[103]
	**Neomycin**	MIP-Gr-MWCNT-/gold electrode	Pyrrole/ Methanol (10%)–Acetic acid (10%)	Direct	Amp	9 × 10^−9^ mol L^−1^ to 7 × 10^−6^ mol L^−1^; 8 × 10^−9^ mol L^−1^	Milk and honey	[104]
	**Chloramphenicol**	MIP/Pt TFME	o-phenylenediamine/0.2 M NaOH/ethanol (4:1, *v*/*v*)	Indirect	SWV	9 × 10^−10^ to 1 × 10^−8^ mol L^−1^; 4 × 10^−10^ mol L^−1^	Honey Milk	[105]
		MIP-NH_2_-Apt [CAP]- AgNP-3-ampy-RGO/GCE	Resorcinol/Buffer solution, acetic acid, ethanol, and acetonitrile at a volume ratio of 2:2:4:2	Indirect	EIS	1 × 10^−12^ to 1 × 10^−9^ mol L^−1^; 3 × 10^−13^ mol L^−1^	Milk	[106]
	**17β-estradiol**	MIP-NPGL/gold electrode	4-Aminothiophenol/ACN-HCl 0.1 M solution (9/1)	Direct	CV	1 × 10^−12^ to 1 × 10^−5^ mol L^−1^; 1 × 10^−13^ mol L^−1^	Water, milk and pork	[107]
	**Olaquindox**	MIP-AuNPs-cMWCNTs/GCE	O-phenylenediamine/methanol/HCl (1:4, *v*/*v*)	Direct	DPV	1 × 10^−9^ to 2 × 10^−6^ mol L^−1^; 3 × 10^−9^ mol L^−1^	Pork Fish	[108]
	**Salbutamol**	MIP-Gr/ PEDOT/SPE	3-aminophenylboronic acid and O-phenylenediamine/0.3 mol L^−1^ H_2_SO_4_	Indirect	DPV	1 × 10^−9^ to 1 × 10^−3^ mol L^−1^; 1 × 10^−9^ mol L^−1^	swine meat and feed samples	[109]
	**Sulfamethoxasole**	MIP-PDA/gold electrode	Dopamine /5% acetic acid	Direct	Amp	8 × 10^−7^ to 2 × 10^−4^ mol L^−1^; 8 × 10^−7^mol L^−1^	Milk	[110]
	**Sulfadimidine**	MIP-NiCo_2_O_4_-3D graphene/GCE	Pyrrole /0.1 M NaOH	Direct	DPV	7 × 10^−13^ to 3 × 10^−9^ mol L^−1^; 6 × 10^−13^ mol L^−1^	Milk	[111]
	**Sulfathiazole**	MIP-CuS-Au-COF/GCE	Pyrrole /0.1 mol L^−1^sodium hydroxide	Direct	DPV	1 × 10^−4^ to 1 × 10^−11^ mol L^−1^; 4 × 10^−12^ mol L^−1^	spiked Fodder and mutton sample chicken liver and pork liver	[112]
**Process and package contaminants**	**Bisphenol A**	MIP-GQDs/GCE	Pyrrole /methanol–acetic acid (8:2, *v*/*v*)	Indirect	DPV	1 × 10^−7^mol L^−1^ to 5 × 10^−5^ mol L^−1^; 4 × 10^−8^ mol L^−1^	Tap and seawater	[113]
		MIP-PBAPolypyrrole/LSG electrode	Pyrrole/methanol–acetic acid (7:3, *v*/*v*)	Direct	DPV	5 × 10^−8^ and 2 × 10^−5^ mol L^−1^; 8 × 10^−9^mol L^−1^.	Tap and mineral water and plastic Samples.	[114]
**Mycotoxin**	**Patulin**	MIP-Thionine-PtNP-NGE/GCE	Thionine/H_2_SO_4_, 2 mol L^−1^	Direct	DPV	1 × 10^−14^ to 1 × 10^−11^ mol L^−1^; 6 × 10^−15^ mol L^−1^	Apple and grape juice	[115]
	**Ochratoxin A**	MIP-MWCNT/GCE	Pyrrole/trimethylamine solution (1%).	Direct	DPV	5 × 10^−8^ to 1 × 10^−6^ mol L^−1^; 4 × 10^−9^ mol L^−1^	Beer and wine	[116]
	**Zearalenone**	MIP-BOMC-IL-Au NPs/GCE	*p*-amino thiophenol/4 M HNO_3_ aqueous solution/methanol (1:1; *v*/*v*)	Direct	SWV	1 × 10^−15^ to 3 × 10^−12^ mol L^−1^; 3 × 10^−16^ mol L^−1^	Corn, Rice and Beer	[117]
	**Deoxynivalenol**	MIP-O-PD/SPGE	*O*-phenylenediamine/50% methanol/acetic acid (9:1, *v*/*v*) + 50% water.	Indirect	EIS	2 × 10^−11^ to 2 × 10^−9^ mol L^−1^; 1 × 10^−12^ mol L^−1^	Cornflakes	[118]
	**Citrinin**	MIP-PdNPs-BZ-GQDs/GCE	Pyrrole/1.0 M NaCl	Direct	DPV	1 × 10^−9^ to 5 × 10^−9^ mol L^−1^; 2 × 10^−10^ mol L^−1^	Chicken egg	[119]
Bacterial contaminants	***Escherichia coli***	MIP/Gold electrode	MAH, HEMA/EGDMA /10 mg/mL lysozyme solution (in 10 mM Tris-HCl buffer, pH 8.0, with 1 mM EDTA)	Indirect	CV	10^2^ to10^7^ CFU/mL; 70 CFU/mL	River water	[120]
	***Staphylococcus epidermidi***	MIP/Gold electrode	3-Aminophenylboronic acid/competitive displacement with fructose and washing with deionized water	Indirect	EIS	10^3^ to 10^7^ CFU/mL; 9.0 nM	No data	[121]

*p*-aminothiophenol (*p*-ATP), Chloramphenicol(CAP), Bisphenol A (PBA), Cypermethrin (CYP),Magnetic nanoparticles(MNPs), *O*-phenylenediamine (OPD), Screen-printed gold electrode (SPGE), pencil graphite electrode (PGE), Chitosan(CS), Nickel cobaltite (NiCo_2_O_4_), Platinum nanoparticles (PtNP), Polyaniline (PANI), Graphene quantum dots (GQDs), Metal–organic frameworks(MOF), Platinum thin-film microelectrode (Pt TFME), Laser-scribed graphene (LSG), *O*-phenylenediamine (OPD), boron-doped ordered mesoporous carbon -gold nanoparticles composite (BOMC-IL-Au NPs), copper sulfide (CuS), Graphene oxide(GO), Reduced Graphene Oxide (RGO), multi-walled carbon nanotubes(MWCNTs), Covalent organic framework(COF), polyhydroxyphenol (PHP), sulfathiazole (STZ), linear sweep voltammetry(LSV), polyaniline(PANI), Screen printed electrode(SPE), single-walled carbon nanotubes(SWCNTs), acrylamide (AM), 2-mercaptonicotinic acid (MNA), Polyoxometalates (POMs), Hierarchical porous dendrimer-like silica nanoparticles (HPSNs-NH2), 3-aminomethyl pyridine (3-Amp), 2-Mercaptobenzothiazole (MBT), 2-hydroxyethyl methacrylate (HEMA).

**Table 3:** MIP-Combined Electrochemical sensors for food sample preparation.

	Analyte/Template	Sensing Configuration	Monomer/Crosslinker/Extraction Solution	Type of Measurement	Detection Technique	LR and LOD	Real Sample	Comment	Ref
**Pesticides**	**Dichlorodiphenyltrichloroethane (DDT)**	MIP-PDA-Fe_3_O_4_/GCE	Dopamine /Acetonitrile	Indirect	EIS	1 × 10^−11^ to 1 × 10^−3^ mol L^−1^;6 × 10^−12^ mol L^−1^	Radish	SPE	[128]
	**Methyl parathion**	MIP/magneto-actuated electrode	MAA/EGDMA/Methanol-acetic acid solution (9:1, *v*/*v*)	Direct	SWV	5 × 10^−6^ to 1 × 10^−4^ mol L^−1^; 5 × 10^−12^ mol L^−1^	Tuna and catfish	MagMIPs	[129]
**Veterinary drugs**	**Fenbendazole**	MIP/Cylindrical carbon fiber microelectrode (CFME)	MAA/EGDMA/Methanol-acetic acid solution (9:1, *v*/*v*)	Direct	SWV	2 × 10^−7^molL^−1^	Beef liver	MISPE	[130]
	**Tetracycline**	MIP-MB/GCE	MAA /Zein/Methanol-acetic acid solution (9:1, *v*/*v*)	Direct	DPV	6 × 10^−11^ to 1 × 10^−6^ mol L^−1^; 6 × 10^−11^mol L^−1^	Milk	MSPE	[131]
**Process and package contaminants**	**Bisphenol A**	MIP-CB-Au/SPE	Monomer: MAA/EGDMA/Methanol-acetic acid solution (9:1, *v*/*v*)	Direct	CV DPV	7 × 10^−8^ to 10 × 10^−6^ mol L^−1^; 9 × 10^−9^ mol L^−1^	Water	SPE	[36]
	**Dibutyl phthalate**	MIP-Fe_3_O_4_-SiO_2_/GCE	MAA /EGDMA /Methanol and acetic acid (volume ratio 6:1)	Indirect	DPV	4 × 10^−11^ to 4 × 10^−6^ mol L^−1^; 2 × 10^−13^ mol L^−1^	Soybean milk And milk	MMISPE	[132]
**Additive**	**Curcumin**	MIP-MB/GCE	MAA /Zein /Methanol-acetic acid solution (9:1, *v*/*v*)	Direct	cv	1 × 10^−7^ to 1 × 10^−4^ mol L^−1^; 1×10^−8^ mol L^−1^	potato chips	MSPE	[133]
**Natural toxin**	**Scombrotoxin**	MIP-Fe_3_O_4_-SiO_2_-MPS//GCE	2-vinyl pyridine /EGDMA /Methanol-acetic acid solution (9:1, *v*/*v*)	Direct	CV	1 × 10^−11^ to 2 × 10^−9^ mol L^−1^; 1 × 10^−11^ mol L^−1^	Fish	MagMIPs	[134]
	**Tyramine**	MIP-PEDOT: PSS AuNP-1-m-4-MP/SPE	MAA /EGDMA/Methanol-acetic acid solution (9:1, *v*/*v*)	Direct	DPV	5 × 10 ^−9^ to 1 × 10^−7^ mol L^−1^; 2 × 10^−9^ mol L^−1^	spiked serum and milk.	sample clean-up and pre-concentration	[135]
**Toxic xenobiotic**	**2,4-dinitrochlorobenzene**	MIP/magneto-actuated electrode	MAA /EGDMA/Ethanol	Direct	DPV	3 × 10^−8^ to 1 × 10^−3^ mol L^−1^; 3 × 10^−8^ mol L^−1^	River and tap water	MagMIPs	[136]

Magnetic molecularly imprinted polymers(MagMIPs), Magnetic nanoparticles(MNPs), Cylindrical carbon fiber microelectrode (CFME), solid-phase extraction (MISPE), magnetic molecularly imprinted solid phase extraction (MMISPE), Gold nanoparticle (AuNP), Poly(3,4-ethylenedioxythiophene) (PEDOT), poly(3,4-ethylenedioxythiophene) polystyrene sulfonate (PEDOT: PSS), 1-methyl-4- mercaptopyridine (1-m-4-MP), Polydopamine (PDA), ethylene glycol maleic rosinate acrylate (EGMRA), 2-hydroxyethyl methacrylate (HEMA).
